# A Review of Modified/Consistent Couple Stress and Strain Gradient Theories for Analyzing Static and Dynamic Behaviors of Functionally Graded Microscale Plates and Shells

**DOI:** 10.3390/ma18194475

**Published:** 2025-09-25

**Authors:** Chih-Ping Wu, Ting-Yu Chang

**Affiliations:** Department of Civil Engineering, National Cheng Kung University, Tainan City 70101, Taiwan; timchang05230523@gmail.com

**Keywords:** dynamic characteristics, functionally graded microscale plates and shells, couple stress theory, strain gradient theory, shear deformation theory, static behavior

## Abstract

This paper provides an overview of various size-dependent theories based on modified/consistent couple stress and strain gradient theories (CSTs and SGTs), highlighting the development of two-dimensional (2D) refined and advanced shear deformation theories (SDTs) and three-dimensional (3D) pure analytical and semi-analytical numerical methods, including their applications, for analyzing the static and dynamic behaviors of microscale plates and shells made from advanced materials such as fiber-reinforced composites, functionally graded (FG) materials, and carbon nanotube/graphene platelet-reinforced composite materials. The strong and weak formulations of the 3D consistent CST, along with their corresponding boundary conditions for FG microplates, are derived and presented for illustration. A comparison study is provided to show the differences in the results of a simply supported FG microplate’s central deflection, stress, and lowest natural frequency obtained using various 2D size-dependent SDTs and 3D analytical and numerical methods based on the consistent CST. A parametric study is conducted to examine how primary factors, such as the effects of dilatational and deviatoric strain gradients and couple stress, impact the static bending and free vibration behaviors of a simply supported FG microplate using a size-dependent local Petrov–Galerkin meshless method based on the consistent SGT. Influences such as the inhomogeneity index and length-to-thickness ratio are considered. It is shown that the significance of the impact of various material length-scale parameters on the central deflection and its lowest natural frequency (in the flexural mode) of the FG microplate is ranked, from greatest to least, as follows: the couple stress effect, the deviatoric strain gradient effect, and finally the dilatational strain gradient effect. Additionally, when the microplate’s thickness is less than 10^−7^ m, the couple stress effect on its static and dynamic behaviors becomes saturated. Conversely, the impact of the dilatational and deviatoric strain gradients consistently influences the microplate’s static and dynamic behaviors.

## 1. Introduction

Functionally graded (FG) material is an emerging industrial material that has been widely used in advanced engineering fields, including aerospace, aeronautical, submarine, electrical, electronic, and communication engineering [1,2,3,4]. Generally, FG material consists of two or more phases of materials with different volume fractions. Because their material properties gradually and smoothly vary within the structural domain, FG structures can eliminate stress concentrations and delamination damage, which often occur in traditional fiber-reinforced composite structures. Additionally, FG structures can achieve specific desirable mechanical properties, including high thermal insulation, a high strength-to-weight ratio, a high stiffness-to-weight ratio, and high corrosion resistance, by optimizing the distribution of the volume fractions of each phase material [5,6,7]. To extend the lifetime of FG structures, developing efficient analytical and numerical methods within the framework of classical continuum mechanics (CCM) and employing them to analyze various mechanical behaviors of FG plate/shell-like structures has gained significant interest. A comprehensive literature review of three-dimensional (3D) and two-dimensional (2D) analytical and numerical methods, along with their applications, can be found in the publications [8,9,10,11,12,13,14,15,16].

Due to the rapid advancements in materials science and manufacturing technology over the past few decades, various components and structures in cutting-edge industries have been miniaturized. However, experimental reports increasingly reveal that when the structure scale approaches the micron level, its mechanical behavior changes significantly from that of macroscale structures. This shift results from size-dependent (or microstructure-dependent) effects caused by notable changes in the microstructure of the constituents. For example, Li et al. [17] and Lei et al. [18] demonstrated through their experiments that the natural frequency of a cantilever nickel microbeam increased by approximately a factor of 2.0 when its thickness decreased from 15 to 2.1 microns. Ince et al. [19] reported that the nominal strength of a concrete specimen increases as its size decreases. Chang et al. [20] found that Young’s modulus of a penta-twinned Ag nanowire increased when its diameter decreased. Using geometrically self-similar indenters, such as cones and pyramids, Pharr et al. [21] observed that the hardness of the specimen increased when the depth of penetration was reduced to less than one micron. In their experimental results, Fleck et al. [22] indicated a significant increase in the torsional hardening of a thin copper wire when its diameter was reduced within the micron-scale range. Lam et al. [23] performed a four-point bending test on an epoxy polymer microbeam, showing a significant increase in its normalized bending rigidity as the microbeam’s thickness decreases within the micron-scale range. McFarland and Colton [24] conducted a static flexural test on a cantilever polypropylene microbeam. They observed notable discrepancies between the experimental results and the predictions obtained from the Euler–Bernoulli beam theory, due to size-dependent effects that occur when the microbeam’s dimensions are reduced within the micron-scale range. These findings indicate that the microstructure’s mechanical behavior is highly dependent on its size. Many classical structural theories based on classical continuum mechanics (CCM) fail to provide precise predictions for the mechanical behavior of microstructures. To address this issue, several higher-order non-CCMs have been developed, including micropolar elasticity [25,26,27], Eringen’s nonlocal elasticity theory (ENET) [28,29,30,31], strain gradient theory (SGT) [23,32,33,34], doublet mechanics [35,36,37], and couple stress theories (CSTs) [38,39,40]. Moreover, Eringen [25] criticized the original CST for having an indeterminacy issue that limited its application. To overcome this, Yang et al. [41] and Hadjesfandiari and Dargush [42,43,44,45] developed the modified CST (MCST) and the consistent CST (CCST), respectively, by deducing that the couple-stress tensor is symmetric and skew-symmetric. This makes MCST and CCST suitable for investigating the various mechanical behaviors of microscale plates and shells, and they are becoming more popular than other non-CCM theories mentioned above because only one material length-scale parameter needs to be calibrated. Subsequently, Yang et al. [41] and Wu and Chang [46] introduced modified and consistent strain gradient theories (MSGT and CSGT), which incorporate the effects of dilatational and deviatoric strain gradients on the MCST and CCST, respectively.

Several review articles on the development of non-CCM theories and their applications have been published [47,48,49,50,51,52,53,54,55,56]. We list these review papers in chronological order, from oldest to newest, in Table 1. These reviews are also categorized based on the theories they explore, the kinematic models they use, the structural types they analyze, and the structural behaviors they examine, helping readers understand the differences among these papers. In this article, we provide a comprehensive review of the development of the MCST, CCST, MSGT, and CSGT, as well as their applications to various mechanical behavior analyses of microscale plates and shells. The current study differs from the review articles mentioned above by focusing on the development of the strong and weak forms of the CCST, MCST, CSGT, and MSGT, along with their relevant 3D semi-analytical and 2D numerical methods, as well as their applications to the static and dynamic behaviors of FG microscale plates and shells, including the significance order for the impact of couple stress and dilatational and deviatoric strain gradients on the static and dynamic behaviors of a simply-supported FG microplate. The purpose of this work is to provide a comprehensive theoretical framework for the CCST, MCST, CSGT, and MSGT, as well as their application scopes, as outlined in the literature. Its layout is organized as follows: In Section 1, we introduce the definition of FG structures, describe the benefits of FG structures, explain size-dependent effects, introduce conventional non-CCM theories, and review relevant existing articles. Section 2 introduces various FG materials, for which the estimates of their effective material properties are presented. In Section 3, we present the strong form of the 3D CCST, including the corresponding Euler–Lagrange equations and associated boundary conditions. We review the relevant 3D analytical and numerical methods based on the strong forms of the 3D MCST, CCST, MSGT, and CSGT, along with their applications to various mechanical behaviors of microscale plates and shells. Additionally, we introduce the weak form of the 3D CCST and review the related semi-analytical numerical methods based on the weak form of the 3D MCST, CCST, MSGT, and CSGT, as well as their applications to different mechanical behaviors of microscale plates and shells. In Section 4, we review several unified 2D size-dependent shear deformation theories (SDTs), based on the MCST, CCST, MSGT, and CSGT, and their applications to various mechanical behaviors of microscale plates and shells. Section 5 conducts a comparative study to illustrate the differences in results obtained using the 3D semi-analytical finite layer method (FLM), the local Petrov–Galerkin meshless (LPGM) method, and 2D advanced and refined SDTs, based on the CCST/MCST. This section also conducts a parametric study to analyze the influence of specific factors on the static and dynamic behaviors of an FG microplate under simply-supported boundary conditions, using the LPGM method based on the CSGT. These factors specifically include the dilatational strain gradient, deviatoric strain gradient, and couple-stress tensors, as well as the inhomogeneity index and the length-to-thickness ratio. Some conclusions from the parametric studies are summarized in Section 6.

## 2. Material Properties

The constituent materials of FG microscale plates and shells cited in the literature can be categorized as exponentially graded (EG) material, power-law FG material, sigmoid FG material, FG carbon nanotube-reinforced composite (CNTRC) material, and FG graphene platelet-reinforced composite (GPLRC) material. The effective material properties of the FG microscale plates and shells mentioned above are presented below.

### 2.1. EG Microscale Plates and Shells

For an EG microscale plate and shell, its material properties are assumed to vary exponentially with the thickness coordinate and are expressed as follows:

(1) $$m_{ij}\left(z\right)=m_{ij}^{b}\;e^{\kappa_{e}\left[\left(z/h\right)+0.5\right]},$$

where the superscript *b* denotes the microstructure’s bottom surface; *z* is the thickness coordinate, with values ranging from −*h*/2 to *h*/2, where *h* is the thickness of the microscale plate or shell being considered; $\kappa_{e}$ is the inhomogeneity index for an EG material, which represents the degree of the material gradient along the thickness and can be determined by the values of the material properties at the top and bottom surfaces, i.e.,

(2) $$\kappa_{e}=\ln{\hat{\kappa }}_{e}=\ln\left(m_{ij}^{t}/m_{ij}^{b}\right),$$

where the superscript *t* represents the microstructure’s top surface; ${\hat{\kappa }}_{e}$ represents the material-property ratio between the top and bottom surfaces.

### 2.2. Power-Law FG Microscale Plates and Shells

For a typical FG microscale plate and shell, its material properties are assumed to follow the power-law distribution of the constituents’ volume fraction along the thickness direction and are given by

(3) $$m_{ij}\left(z\right)=m_{ij}^{t}\Gamma \left(z\right)+m_{ij}^{b}\left[1-\Gamma \left(z\right)\right],$$

where the symbol Γ(z) denotes the volume fraction of the material at the top surface and is defined as $\Gamma \left(z\right)={\left[\left(z/h\right)+0.5\right]}^{k_{p}}$; $k_{p}$ is the inhomogeneity index for a power-law FG material. When $k_{p}$ = 0 and $k_{p}$ = ∞, the FG microscale plate and shell reduce to a homogeneous microscale plate and shell with material properties $m_{ij}^{t}$ and $m_{ij}^{b}$, respectively.

### 2.3. Sigmoid FG Microscale Plates and Shells

For a sigmoid FG microscale plate and shell, the material properties are assumed to follow the sigmoid function of the constituents’ volume fractions through the thickness direction and are expressed as follows:

(4a) $$m_{ij}\left(z\right)=1-\left(1/2\right){\left\{\left[\left(h/2\right)-z\right]/\left(h/2\right)\right\}}^{\kappa_{s}}\;\mathrm{when}\;0<z<\left(h/2\right),$$

(4b) $$m_{ij}\left(z\right)=\left(1/2\right){\left\{\left[\left(h/2\right)+z\right]/\left(h/2\right)\right\}}^{\kappa_{s}}\;\mathrm{when}\;-\left(h/2\right)<z<0,$$

where $k_{s}$ is the inhomogeneity index for a sigmoid FG material.

### 2.4. FG-CNTRC Microscale Plates and Shells

Because CNTs have exceptional chemical, physical, and electrical properties, they have been incorporated into the polymer matrix to create the FG-CNTRC material. Five different distribution functions of CNTs, varying in the thickness direction, are commonly discussed in the literature: uniformly distributed (UD), as well as FG A-, O-, V-, and X-type variations.

The through-thickness distributions of the volume fraction of CNTs, $\Gamma_{CNT}$, for the five aforementioned types of FG-CNTRC microscale plates and shells are presented as follows:

(5a) $$\Gamma_{CNT}=\Gamma_{CNT}^{*}\;\;\;(\mathrm{UD}\text{-}\mathrm{type}\;\mathrm{variation}),$$

(5b) $$\Gamma_{CNT}\left(z\right)=\left[1-\left(2z\right)/h\right]\Gamma_{CNT}^{*}\;\;\;(\mathrm{FG}A\text{-}\mathrm{type}\;\mathrm{variation}),$$

(5c) $$\Gamma_{CNT}\left(z\right)=2\left[1-\left(2\left|z\right|/h\right)\right]\Gamma_{CNT}^{*}\;\;\;(\mathrm{FG}O\text{-}\mathrm{type}\;\mathrm{variation}),$$

(5d) $$\Gamma_{CNT}\left(z\right)=\left[1-\left(2z\right)/h\right]\Gamma_{CNT}^{*}\;\;\;(\mathrm{FG}V\text{-}\mathrm{type}\;\mathrm{variation}),$$

(5e) $$\Gamma_{CNT}\left(z\right)=2\left(2\left|z\right|/h\right)\Gamma_{CNT}^{*}\;\;\;(\mathrm{FG}X\text{-}\mathrm{type}\;\mathrm{variation}),$$

where $\Gamma_{CNT}^{*}$ denotes the volume fraction index of CNTs and is expressed as

(6) $$\Gamma_{CNT}^{*}=W_{CNT}/\left[W_{CNT}+\left(\rho_{CNT}/\rho_{m}\right)-\left(\rho_{CNT}/\rho_{p}\right)W_{CNT}\right],$$

where $W_{CNT}$ represents the mass fraction of CNTs in the FG-CNTRC microstructure, and $\rho_{CNT}$ and $\rho_{p}$ are the mass densities of the CNTs and the polymer matrix, respectively.

The rule of mixtures [57] has been applied to estimate the effective material properties of an FG-CNTRC microscale plate and shell, which vary through the thickness and are presented as follows.

(7a) $$E_{11}=\lambda_{1}\Gamma_{CNT}{\left(E_{11}\right)}_{CNT}+\left(\Gamma_{p}E_{p}\right),$$

(7b) $$\left(\lambda_{2}/E_{22}\right)=\Gamma_{CNT}/{\left(E_{22}\right)}_{CNT}+\left(\Gamma_{p}/E_{p}\right),$$

(7c) $$\left(\lambda_{3}/G_{12}\right)=\Gamma_{CNT}/{\left(G_{12}\right)}_{CNT}+\left(\Gamma_{p}/G_{p}\right),$$

where ${\left(E_{11}\right)}_{CNT}$, ${\left(E_{22}\right)}_{CNT}$, and ${\left(G_{12}\right)}_{CNT}$ represent Young’s moduli and shear modulus of CNTs; $E_{p}$ and $G_{p}$ represent Young’s moduli and shear modulus of the polymer matrix; $\lambda_{i}$ (i=1−3) are the CNT efficiency parameters, which are determined by equalizing the material properties of the FG-CNTRC material obtained using the rule of mixtures and molecular dynamics simulation. Shen and Xiang [57] indicated that the value ranges of $\lambda_{i}$ are related to the value of $\Gamma_{CNT}^{*}$. For a polymer matrix, when $\Gamma_{CNT}^{*}=0.11$, the values of $\lambda_{1},\;\lambda_{2},\;\mathrm{and}\;\lambda_{3}$ are reported in the literature as 0.149, 0.934, and 0.934; these values are 0.150, 0.941, and 0.941 for the case of $\Gamma_{CNT}^{*}=0.14$ and 0.149, 1.381, and 1.381 for the case of $\Gamma_{CNT}^{*}=0.17$. $\Gamma_{CNT}$ and $\Gamma_{p}$ are the volume fractions of CNTs and the polymer matrix, respectively, in which $\Gamma_{CNT}+\Gamma_{p}=1$.

The Poisson’s ratio $\nu_{12}$ of the FG-CNTRC material is determined in the same way, as follows:

(8) $$\nu_{12}=\Gamma_{CNT}^{*}{\left(\nu_{12}\right)}_{CNT}+\left(\Gamma_{p}\nu_{p}\right),$$

where ${\left(\nu_{12}\right)}_{CNT}$ and $\nu_{p}$ are Poisson’s ratios of the CNTs and the polymer matrix, respectively. $\nu_{12}$ is considered as a constant through the thickness coordinate of the FG-CNTRC material, and $\nu_{12}=\nu_{13}=\nu_{23}$.

By utilizing Equations (7a–c) and (8), we can derive the through-thickness distributions of the effective properties of the FG-CNTRC microstructures.

### 2.5. FG-GPLRC Microscale Plates and Shells

An alternative nanomaterial, GPL, exhibits exceptional chemical, physical, and electrical properties. As a result, it has been incorporated into the polymer matrix to create the FG-GPLRC material. Consequently, five GPL distribution patterns, varying in the thickness direction, have been examined in the relevant literature, including UD and FG A-type, O-type, V-type, and X-type variations. Furthermore, the Halpin–Tsai model [58] is used to estimate the effective Young’s modulus, while the rule of mixtures [58] is applied to calculate the effective Poisson’s ratio and effective mass density. As a result, the following equations are used to describe these material properties.

According to the Halpin–Tsai model, the effective Young’s modulus of the FG-GPLRC microscale plate or shell $E_{eff}$ can be approximated with

(9) $$E_{eff}=\left(3/8\right)E_{L}+\left(5/8\right)E_{T},$$

where $E_{L}$ and $E_{T}$ denote the longitudinal and transverse moduli, respectively, and they are expressed following Song et al. [59] as follows:

(10a) $$E_{L}=\left[\left(1+\xi_{L}\eta_{L}V_{GPL}\right)/\left(1-\eta_{L}V_{GPL}\right)\right]E_{p},$$

(10b) $$E_{T}=\left[\left(1+\xi_{T}\eta_{T}V_{GPL}\right)/\left(1-\eta_{T}V_{GPL}\right)\right]E_{p},$$

(10c) $$\eta_{L}=\left[\left(E_{GPL}/E_{p}\right)-1\right]/\left[\left(E_{GPL}/E_{p}\right)+\xi_{L}\right],$$

(10d) $$\eta_{T}=\left[\left(E_{GPL}/E_{p}\right)-1\right]/\left[\left(E_{GPL}/E_{p}\right)+\xi_{T}\right],$$

(10e) $$\xi_{L}=2\left[{\left(L_{x}\right)}_{GPL}/h_{GPL}\right],$$

(10f) $$\xi_{T}=2\left[{\left(L_{y}\right)}_{GPL}/h_{GPL}\right],$$

where $E_{GPL}$ denotes Young’s modulus of the GPLs; $V_{\mathrm{GPL}}$ is the volume fraction of the GPLs; $\xi_{L}\;\mathrm{and}\;\xi_{T}$ are the parameters characterizing the geometrical dimensions of the GPLs; $\eta_{L}\;\mathrm{and}\;\eta_{T}$ are the parameters describing the geometrical dimensions of the GPLs and Young’s modulus ratio between the GPLs and the polymer matrix; and ${\left(L_{x}\right)}_{GPL},\;\;{\left(L_{y}\right)}_{GPL},\;\mathrm{and}\;h_{GPL}$ represent the length, width, and thickness of the GPLs, respectively. In a parametric study [59], the size ranges of the geometric parameters of the GPLs are given as $1\;\leq \;{\left(L_{x}\right)}_{GPL}/\;\;{\left(L_{y}\right)}_{GPL}\;\leq \;3$, and $20\;\leq \;{\left(L_{x}\right)}_{GPL}/\;\;h_{GPL}\leq \;2000$. A specific set of geometric parameters, $\;{\left(L_{x}\right)}_{GPL}=2.5\times 10^{-6}\;m,$ $\;{\left(L_{y}\right)}_{GPL}=1.5\times 10^{-6}\;m,$ and $\;h_{GPL}=1.5\times 10^{-9}\;m,$ was commonly used in the relevant literature.

Following the rule of mixtures, we express the effective Poisson’s ratio $\upsilon_{eff}$ and the effective mass density $\rho_{eff}$ of the GPLRC material as follows:

(11) $$\upsilon_{eff}=\upsilon_{p}+\left(\upsilon_{GPL}-\upsilon_{p}\right)V_{GPL},$$

(12) $$\rho_{eff}=\rho_{p}+\left(\rho_{GPL}-\rho_{p}\right)V_{GPL},$$

where the subscripts *GPL* and *eff* denote the GPLs and the GPLRC material, respectively; $V_{GPL}$ represents the volume fractions of the GPLs. In the numerical example, with a specific value of $W_{GPL}^{*}$, the weight fractions of five relevant distribution patterns of the GPLs varying through the thickness of the microscale shell ($W_{GPL}\left(\zeta \right)$) are expressed as follows:

(13a) $$W_{GPL}=W_{GPL}^{*}\;\;\;(\mathrm{UD}\text{-}\mathrm{type}\;\mathrm{variation}),$$

(13b) $$W_{GPL}\left(\zeta \right)=\left[1-\left(2\zeta /h\right)\right]W_{GPL}^{*}\;\;\;(\mathrm{FG}A\text{-}\mathrm{type}\;\mathrm{variation}),$$

(13c) $$W_{GPL}\left(\zeta \right)=2\left[1-\left(2\left|\zeta \right|/h\right)\right]W_{GPL}^{*}\;\;\;(\mathrm{FG}O\text{-}\mathrm{type}\;\mathrm{variation}),$$

(13d) $$W_{GPL}\left(\zeta \right)=\left[1+\left(2\zeta /h\right)\right]W_{GPL}^{*}\;\;\;(\mathrm{FG}V\text{-}\mathrm{type}\;\mathrm{variation}),$$

(13e) $$W_{GPL}\left(\zeta \right)=2\left(2\left|\zeta \right|/h\right)W_{GPL}^{*}\;\;\;(\mathrm{FG}X\text{-}\mathrm{type}\;\mathrm{variation}),$$

where the total volume fraction of the GPLs in each case, given in Equations (13a)–(13e), can be obtained using the relationship between $V_{GPL}$ and $W_{GPL}$, which is $V_{GPL}=W_{GPL}/\left[W_{GPL}+\left(\rho_{GPL}/\rho_{p}\right)\left(1-W_{GPL}\right)\right].$

## 3. Consistent Couple Stress Theory

### 3.1. Strong Form

As mentioned above, Hadjesfandiari and Dargush [42,43] reformulated the original CST by deducing that the couple-stress tensor is skew-symmetric and subsequently proposed the CCST. Within the framework of the CCST, we employ Hamilton’s principle to derive an alternative version of the strong form of the CCST by selecting the displacements and transverse shear and normal stresses as the primary variables subjected to variation. The Euler–Lagrange equations and associated boundary conditions are presented below. By incorporating specific interpolation or approximation functions into the mixed strong form of the 3D CCST, a semi-analytical point collocation method can be developed for analyzing the mechanical behaviors of FG microscale plates and shells.

#### 3.1.1. Fundamental Equations

**Figure 1:**
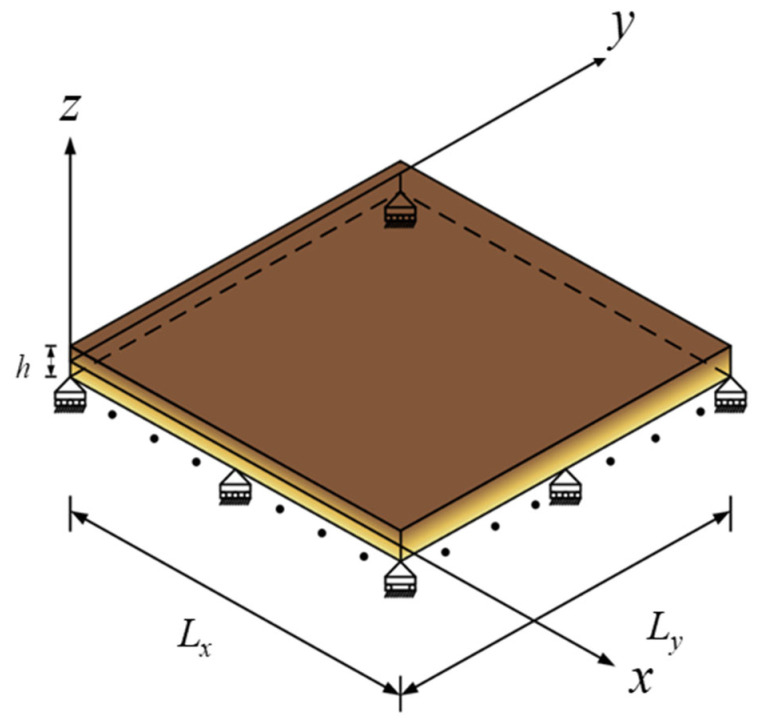
Schematic diagram of a simply supported FG elastic microplate.

We consider an elastic isotropic FG microplate, as illustrated in Figure 1. A Cartesian coordinate system, consisting of *x*, *y*, and *z* coordinates, is oriented such that the *xy*-plane serves as its central plane. The length, width, and thickness of the microplate are defined as *L_x_*, *L_y_*, and *h*, respectively.

In CCST, Hadjesfandiari and Dargush indicated that, generally speaking, due to the size-dependent effect, when an elastic microscale solid is subjected to applied external loads, the induced force-stress and couple-stress tensors should be asymmetric and skew-symmetric, respectively.

Hadjesfandiari and Dargush distinguished the force-stress and couple-stress tensors by using parentheses and brackets to enclose a pair of indices. Additionally, Hadjesfandiari and Dargush derived a relationship between the skew-symmetric part of the force-stress tensor and the couple-stress tensor, which was expressed as [42,43]

(14) $$\sigma_{\left[ij\right]}=\mu_{\left[i,j\right]}=\left(1/2\right)\left(\partial \mu_{i}/\partial j\right)-\left(\partial \mu_{j}/\partial i\right)\;\;\;\left(i,\;j=x,\;y,\;\mathrm{and}\;z\right),$$

where $\sigma_{\left[ij\right]}$ denotes the skew-symmetric part of the force-stress tensor; and $\mu_{i}$ is the couple-stress tensor, such that $\mu_{i}=\mu_{kj}=-\mu_{jk}$, and the indices *i*, *j*, and *k* are followed in a right-hand screw rule order.

Based on Equation (14), Hadjesfandiari and Dargush derived a consistent differential operator for the force-stress tensor in classical elasticity theory and the total force-stress tensor in the CCST.

For a typical isotropic microplate, the linear constitutive equations are expressed as follows [42,43]:

(15) $$\left\{\begin{array}{c} \sigma_{\left(xx\right)}\\ \sigma_{\left(yy\right)}\\ \sigma_{\left(zz\right)}\\ \sigma_{\left(xz\right)}\\ \sigma_{\left(yz\right)}\\ \sigma_{\left(xy\right)} \end{array}\right\}=\left[\begin{array}{cccccc} c_{11} & c_{12} & c_{12} & 0 & 0 & 0\\ c_{12} & c_{11} & c_{12} & 0 & 0 & 0\\ c_{12} & c_{12} & c_{11} & 0 & 0 & 0\\ 0 & 0 & 0 & c_{66} & 0 & 0\\ 0 & 0 & 0 & 0 & c_{66} & 0\\ 0 & 0 & 0 & 0 & 0 & c_{66} \end{array}\right]\;\left\{\begin{array}{c} \varepsilon_{xx}\\ \varepsilon_{yy}\\ \varepsilon_{zz}\\ 2\varepsilon_{xz}\\ 2\varepsilon_{yz}\\ 2\varepsilon_{xy} \end{array}\right\},$$

(16) $$\left\{\begin{array}{c} \mu_{x}\\ \mu_{y}\\ \mu_{z} \end{array}\right\}=-2Gl^{2}\left\{\begin{array}{c} \kappa_{x}\\ \kappa_{y}\\ \kappa_{z} \end{array}\right\},$$

where $\sigma_{\left(xx\right)},\;\;\sigma_{\left(yy\right)},\;\ldots ,\;\mathrm{and}\;\sigma_{\left(xy\right)}$ represent the symmetric part of the force-stress components, i.e., $\sigma_{\left(ij\right)}=\sigma_{\left(ji\right)}$; $\varepsilon_{xx},\;\;\varepsilon_{yy},\;\ldots ,\;\;\mathrm{and}\;\varepsilon_{xy}$ are the strain components; $\kappa_{i}$ is the skew-symmetric part of the curvature tensor, such that $\kappa_{i}=\kappa_{kj}=-\kappa_{jk}$; *c_ij_* represents the elastic coefficients, and $c_{11}=E\left(1-\upsilon \right)/\left[\left(1+\upsilon \right)\left(1-2\upsilon \right)\right],$ $c_{12}=\left(E\upsilon \right)/\left[\left(1+\upsilon \right)\left(1-2\upsilon \right)\right],$ and $c_{66}=G$, where E, υ, and G represent Young’s modulus, Poisson’s ratio, and shear modulus. For comparison purposes, it is noted that we revise the coupling couple stress–skew-symmetric part of the curvature coefficient from $-8G{\hat{l}}^{2}$ to $-2Gl^{2}$, where $l\;\mathrm{and}\;\hat{l}$ represent the material length-scale parameters for the MCST and CCST, respectively, for which $\hat{l}=l/2$.

The strain tensor is defined as $\varepsilon =\left(1/2\right)\left[\nabla u+{\left(\nabla u\right)}^{T}\right],$ such that the relationship between the strain tensor and the displacement tensor is expressed as follows [42,43]:

(17) $$\left\{\begin{array}{c} \varepsilon_{xx}\\ \varepsilon_{yy}\\ \varepsilon_{zz}\\ 2\varepsilon_{yz}\\ 2\varepsilon_{xz}\\ 2\varepsilon_{xy} \end{array}\right\}=\left[\begin{array}{ccc} \partial_{x} & 0 & 0\\ 0 & \partial_{y} & 0\\ 0 & 0 & \partial_{z}\\ 0 & \partial_{z} & \partial_{y}\\ \partial_{z} & 0 & \partial_{x}\\ \partial_{y} & \partial_{x} & 0 \end{array}\right]\;\left\{\begin{array}{c} u_{x}\\ u_{y}\\ u_{z} \end{array}\right\},$$

where $\partial_{i}=\partial /\partial i$, i=x , y  and  z.

The rotation tensor is defined as $\Omega_{i}=\Omega_{kj}=-\Omega_{jk}$ and $\Omega =\left(1/2\right)\mathrm{Curl}u$, such that the relationship between the rotation tensor and displacement tensor is expressed as follows [42,43]:

(18) $$\left\{\begin{array}{c} \Omega_{x}\\ \Omega_{y}\\ \Omega_{z} \end{array}\right\}=\left(1/2\right)\left[\begin{array}{ccc} 0 & -\partial_{z} & \partial_{y}\\ \partial_{z} & 0 & -\partial_{x}\\ -\partial_{y} & \partial_{x} & 0 \end{array}\right]\;\left\{\begin{array}{c} u_{x}\\ u_{y}\\ u_{z} \end{array}\right\}.$$

The symmetric part of the curvature tensor is defined as $\chi_{kj}=\chi_{jk}$ and $\chi =\left(1/2\right)\left[\nabla \Omega +{\left(\nabla \Omega \right)}^{T}\right],$ such that the relationship between the symmetric part of the curvature tensor and the displacement tensor is expressed as follows [42,43]:

(19) $$\left\{\begin{array}{c} \chi_{xx}\\ \chi_{yy}\\ \chi_{zz}\\ \chi_{yz}\\ \chi_{xz}\\ \chi_{xy} \end{array}\right\}=\left(1/4\right)\left[\begin{array}{ccc} 0 & -2\partial_{xz} & 2\partial_{xy}\\ 2\partial_{yz} & 0 & -2\partial_{xy}\\ -2\partial_{yz} & \partial_{xz} & 0\\ \left(-\partial_{yy}+\partial_{zz}\right) & \partial_{xy} & -\partial_{xz}\\ -\partial_{xy} & \left(\partial_{xx}-\partial_{zz}\right) & \partial_{yz}\\ \partial_{xz} & -\partial_{yz} & \left(-\partial_{xx}+\partial_{yy}\right) \end{array}\right]\;\left\{\begin{array}{c} u_{x}\\ u_{y}\\ u_{z} \end{array}\right\}.$$

As mentioned above, the skew-symmetric part of the curvature tensor is defined as $\kappa_{i}=\kappa_{kj}=-\kappa_{jk}$, and $\kappa =\left(1/2\right)\mathrm{Curl}\Omega$, such that the relationship between the skew-symmetric part of the curvature tensor and the displacement tensor is expressed as follows [42,43]:

(20) $$\left\{\begin{array}{c} \kappa_{x}\\ \kappa_{y}\\ \kappa_{z} \end{array}\right\}=\left(1/4\right)\left[\begin{array}{ccc} \left(-\partial_{yy}-\partial_{zz}\right) & \;\partial_{xy} & \;\partial_{xz}\\ \partial_{xy} & \;\left(-\partial_{xx}-\partial_{zz}\right) & \;\partial_{yz}\\ \partial_{xz} & \;\partial_{yz} & \;\left(-\partial_{xx}-\partial_{yy}\right) \end{array}\right]\;\left\{\begin{array}{c} u_{x}\\ u_{y}\\ u_{z} \end{array}\right\}.$$

#### 3.1.2. Hamilton’s Principle

Hamilton’s principle is utilized to derive the Euler–Lagrange equations for the microplate, along with its possible boundary conditions. The corresponding energy functional is expressed as follows:

(21) $$I=\int_{t_{1}}^{t_{2}}L\;dt,$$

where *L* denotes the Lagrange functional, which is defined as follows [60]:

(22) $$L=T-\Pi_{R}+W,$$

where *T*, $\Pi_{R}$, and *W* represent the kinetic energy and Reissner’s strain energy of the microplate and the work resulting from the action of the bi-axial compression, and are expressed as follows:

(23) $$T=\int_{-h/2}^{h/2}\iint_{A}\left(\rho /2\right)\left[{\left(\partial u_{x}/\partial t\right)}^{2}+{\left(\partial u_{y}/\partial t\right)}^{2}+{\left(\partial u_{z}/\partial t\right)}^{2}\right]dxdydz,$$

(24) $$\begin{array}{l} \Pi_{R}=\int_{-h/2}^{h/2}\iint_{A}\left[\sigma_{\left(xx\right)}\varepsilon_{xx}+\sigma_{\left(yy\right)}\varepsilon_{yy}+\sigma_{\left(zz\right)}\varepsilon_{zz}+2\sigma_{\left(xz\right)}\varepsilon_{xz}+2\sigma_{\left(yz\right)}\varepsilon_{yz}+2\sigma_{\left(xy\right)}\varepsilon_{xy}\right.\\ \;\;-B\left(\sigma_{\left(xy\right)},\;\mu_{k}\right)\left.-2\mu_{zy}\kappa_{zy}-2\mu_{xz}\kappa_{xz}-2\mu_{yx}\kappa_{yx}\right]dxdydz-\iint_{A^{+}}{\bar{q}}_{z}\left(x,\theta \right)u_{z}^{+}dxdy\\ \;-\int_{-h/2}^{h/2}\int_{\Gamma_{\sigma }}\left({\bar{t}}_{x}u_{x}+{\bar{t}}_{y}u_{y}+{\bar{t}}_{z}u_{z}+{\bar{m}}_{x}\Omega_{x}+{\bar{m}}_{y}\Omega_{y}+{\bar{m}}_{z}\Omega_{z}\right)d\Gamma dz-\int_{-h/2}^{h/2}\int_{\Gamma_{u}}\left[(u_{x}-{\bar{u}}_{x})t_{x}\right.\\ \;\left.+(u_{y}-{\bar{u}}_{y})t_{y}+(u_{z}-{\bar{u}}_{z})t_{z}+\left(\Omega_{x}-{\bar{\Omega }}_{x}\right)m_{x}+\left(\Omega_{y}-{\bar{\Omega }}_{y}\right)m_{y}+\left(\Omega_{z}-{\bar{\Omega }}_{z}\right)m_{z}\right]d\Gamma dz, \end{array}$$

(25) $$W=-\iint_{A^{+}}\bar{q}w^{+}dxdy\;+\iint_{A}\left({\bar{N}}_{xx}^{0}\;\varepsilon_{xx}^{nl}+{\bar{N}}_{yy}^{0}\;\varepsilon_{yy}^{nl}\right)dxdy,$$

where *A* represents the microplate domain on the xy-plane, and $A^{+}$ represents the microplate’s top surface at z=h/2, upon which the transverse loads (${\bar{q}}_{z}$) are applied; $\Gamma_{\sigma }$ and $\Gamma_{u}$ represent the portions of the edge boundary, where the surface traction force and moment components and the displacement and rotation components are prescribed, respectively; B(σ, μ) is the complementary energy density function, in which $\varepsilon_{ij}=\partial B/\partial \sigma_{\left(ij\right)}$ and $\kappa_{kl}=-\partial B/\partial \mu_{kl}$; ${\bar{N}}_{xx}^{0}\;\mathrm{and}\;{\bar{N}}_{yy}^{0}$ are the applied resultant forces, and $\varepsilon_{xx}^{nl}\;\mathrm{and}\;\varepsilon_{yy}^{nl}$ are the von Kármán second-order strains and are expressed as $\varepsilon_{xx}^{nl}=\left(1/2\right){\left(\partial u_{z}/\partial x\right)}^{2}$ and $\varepsilon_{yy}^{nl}=\left(1/2\right){\left(\partial u_{z}/\partial y\right)}^{2}$.

In the mixed formulation, the elastic displacement and transverse force–stress components are taken as the primary variables subject to variation.

#### 3.1.3. Euler–Lagrange Equations and Possible Boundary Conditions

Substituting Equations (23)–(25) into Equation (21), employing Hamilton’s principle (i.e., δI=0), and conducting integration by parts using Green’s theorem, we can finally obtain the Euler–Lagrange equations of the 3D CCST from the resulting domain integral terms and the possible boundary conditions from the resulting boundary integral terms, which are expressed below.

The Euler–Lagrange equations are expressed as follows:

(26) $$\delta u_{x}:\;-\sigma_{\left(xx\right)}{,}_{x}-\sigma_{\left(yx\right)}{,}_{y}-\tau_{\left(zx\right)}{,}_{z}+\left(1/2\right)\mu_{x}{,}_{yy}-\left(1/2\right)\mu_{y}{,}_{xy}+\left(1/2\right)\mu_{x}{,}_{zz}-\left(1/2\right)\mu_{z}{,}_{xz}=-\rho u_{x}{,}_{tt},$$

(27) $$\delta u_{y}:-\sigma_{\left(xy\right)}{,}_{x}-\sigma_{\left(yy\right)}{,}_{y}-\tau_{\left(zy\right)}{,}_{z}-\left(1/2\right)\mu_{x}{,}_{xy}+\left(1/2\right)\mu_{y}{,}_{xx}+\left(1/2\right)\mu_{y}{,}_{zz}-\left(1/2\right)\mu_{z}{,}_{yz}=-\rho u_{y}{,}_{tt},$$

(28) $$\delta u_{z}:\begin{array}{l} -\sigma_{\;\left(xz\right)}{,}_{x}-\sigma_{\left(yz\right)}{,}_{y}-\sigma_{\left(zz\right)}{,}_{z}-\left(1/2\right)\mu_{x}{,}_{xz}+\left(1/2\right)\mu_{z}{,}_{xx}-\left(1/2\right)\mu_{y}{,}_{zy}+\left(1/2\right)\mu_{z}{,}_{yy}\\ \;\;\;\;\;\;\;\;\;\;\;\;\;\;=-\rho u_{z}{,}_{tt}-\left({\bar{N}}_{xx}/h\right)u_{z}{,}_{xx}-\left({\bar{N}}_{yy}/h\right)u_{z}{,}_{yy}, \end{array}$$

(29) $$\delta \sigma_{\left(xz\right)}:\;u_{x}{,}_{z}+u_{z}{,}_{x}-c_{55}^{-1}\sigma_{\left(xz\right)}=0,$$

(30) $$\delta \sigma_{\left(yz\right)}:\;u_{y}{,}_{z}+u_{y}{,}_{z}-c_{44}^{-1}\sigma_{\left(yz\right)}=0,$$

(31) $$\delta \sigma_{\left(zz\right)}:\;u_{z}{,}_{z}+\left(c_{12}/c_{11}\right)u_{x}{,}_{x}+\left(c_{12}/c_{11}\right)u_{y}{,}_{y}-c_{33}^{-1}\sigma_{\left(zz\right)}=0,$$

The surface boundary conditions are obtained as follows:

(32a) $$\left[\begin{array}{ccccc} \sigma_{zx} & \sigma_{zy} & \sigma_{zz} & \mu_{zx} & \mu_{zy} \end{array}\right]=\left[\;\begin{array}{ccccc} 0 & 0 & {\bar{q}}_{z} & 0 & 0 \end{array}\right]\;\mathrm{on}\;z=h/2,$$

(32b) $$\left[\begin{array}{ccccc} \sigma_{zx} & \sigma_{zy} & \sigma_{zz} & \mu_{zx} & \mu_{zy} \end{array}\right]=\left[\;\begin{array}{ccccc} 0 & 0 & 0 & 0 & 0 \end{array}\right]\;\mathrm{on}\;z=-h/2;$$

The possible boundary conditions are obtained as follows:

(33a) $$\sigma_{\left(xx\right)}n_{x}+\left[\sigma_{\left(xy\right)}-\left(1/2\right)\mu_{zy}{,}_{y}+\left(1/2\right)\mu_{xz}{,}_{x}\right]n_{y}={\bar{t}}_{x}\;\mathrm{or}\;u_{x}={\bar{u}}_{x},$$

(33b) $$\left[\sigma_{\left(xy\right)}+\left(1/2\right)\mu_{zy}{,}_{y}-\left(1/2\right)\mu_{xz}{,}_{x}\right]n_{x}+\sigma_{\left(yy\right)}n_{y}={\bar{t}}_{y}\mathrm{or}\;u_{y}={\bar{u}}_{y},$$

(33c) $$\begin{array}{l} \left[\sigma_{\left(xz\right)}+\left(1/2\right)\mu_{zy}{,}_{z}-\left(1/2\right)\mu_{yx}{,}_{x}-\left({\bar{N}}_{xx}/h\right)u_{z}{,}_{x}\right]n_{x}\\ \;\;\;+\left[\sigma_{\left(yz\right)}+\left(1/2\right)\mu_{xz}{,}_{z}-\left(1/2\right)\mu_{yx}{,}_{y}-\left({\bar{N}}_{yy}/h\right)u_{z}{,}_{y}\right]n_{y}={\bar{t}}_{z} \end{array}\mathrm{or}\;u_{z}={\bar{u}}_{z},$$

(33d) $$\mu_{yx}n_{y}={\bar{m}}_{x}\;\;\mathrm{or}\;\;\Omega_{x}={\bar{\Omega }}_{x},$$

(33e) $$\mu_{yx}n_{x}={\bar{m}}_{y}\;\;\mathrm{or}\;\;\Omega_{y}={\bar{\Omega }}_{y},$$

(33f) $$\mu_{xz}n_{x}+\mu_{zy}n_{y}={\bar{m}}_{z}\;\;\mathrm{or}\;\;\Omega_{z}={\bar{\Omega }}_{z},$$

where $n_{x}$ and $n_{y}$ represent the components of the unit normal vectors on the edges.

The Euler–Lagrange Equations (26)–(31), along with the possible boundary condition (33a–f), constitute a well-defined mathematical problem and are capable of investigating the static bending, free vibration, and static buckling behaviors of microplates. By incorporating the differential reproducing kernel (DRK) interpolants [61] into the strong form of the CCST, Wu and Chang [62] and Wu and Chou [63] developed a semi-analytical meshless point collocation method to evaluate the static bending, static buckling, and free vibration behaviors of FG microplates. Utilizing the perturbation method, Wu and Lyu [64] presented an asymptotic theory for investigating the free vibration characteristics of FG microplates. On the other hand, based on the strong form of the 3D MCST and utilizing the state-space analytical method, Salehipour et al. [65,66] presented exact closed-form solutions for the free vibration analysis of FG microplates, as well as the static bending and free vibration analyses of porous FG cylindrical microscale shells. Based on the strong form of the MSGT, Salehipour and Shahsavar [67] provided the 3D solutions for the free vibration analysis of FG microplates. The aforementioned 3D analyses [62,63,64,65,66,67], within the framework of the 3D CCST, MCST, and MSGT, are rare and valuable because they provide a reference for assessing the accuracy of size-dependent 2D advanced and refined SDTs. They are listed in Table 2 in chronological order, from oldest to most recent.

### 3.2. Weak Form

#### 3.2.1. Hamilton’s Principle

A weak formulation of the 3D CCST can also be derived using the Hamilton principle, for which the displacements are taken as the primary variables subject to variation. Conducting the first-order variation of the kinetic energy, strain energy, and work performed, utilizing the kinematics assumptions provided in Equations (23)–(25), and performing integration by parts, we finally express the resulting equations as follows:

(34) $$\delta U_{s}=\iint_{A}\int_{-h/2}^{h/2}\left\{{\left(\delta \varepsilon_{n}\right)}^{T}\sigma_{n}+{\left(\delta \varepsilon_{s}\right)}^{T}\sigma_{s}-2{\left(\delta \kappa \right)}^{T}\mu \right\}dxdydz,$$

(35) $$\delta T=-\iint_{A}\int_{-h/2}^{h/2}\left[\rho {\left(\delta u\right)}^{T}u{,}_{tt}+\rho {\left(\delta w\right)}^{T}w{,}_{tt}\right]dxdydz,$$

(36) $$\delta W=-\iint_{A^{+}}\bar{q}\delta w^{+}dxdy+\iint_{A}\left({\bar{N}}_{xx}^{0}\;\delta \varepsilon_{xx}^{nl}+{\bar{N}}_{yy}^{0}\;\delta \varepsilon_{yy}^{nl}\right)dxdy,$$

where the superscript *T* represents the transposition of the matrices or vectors,

(37a) $$\varepsilon_{n}={\left[\begin{array}{ccc} \varepsilon_{xx} & \varepsilon_{yy} & \varepsilon_{zz} \end{array}\right]}^{T}=B_{1}u+B_{2}w,$$

(37b) $$\varepsilon_{s}={\left[\begin{array}{cc} \gamma_{yz} & \;\gamma_{xz}\;\gamma_{xy} \end{array}\right]}^{T}=B_{3}u+B_{4}w,$$

(37c) $$\kappa ={\left[\begin{array}{cc} \kappa_{x} & \;\kappa_{y}\;\kappa_{z} \end{array}\right]}^{T}=B_{5}u+B_{6}w,$$

(37d) $$u=\left[\begin{array}{c} u\\ v \end{array}\right],$$

(37e) $$\sigma_{n}={\left[\begin{array}{cc} \sigma_{\left(xx\right)} & \;\sigma_{\left(yy\right)}\;\sigma_{\left(zz\right)} \end{array}\right]}^{T}=Q_{cn}B_{1}u+Q_{cn}B_{2}w,$$

(37f) $$\sigma_{s}={\left[\begin{array}{cc} \sigma_{yz} & \;\sigma_{xz}\;\sigma_{xy} \end{array}\right]}^{T}=Q_{cs}B_{3}u+Q_{cs}B_{4}w,$$

(37g) $$\mu ={\left[\begin{array}{ccc} \mu_{x} & \mu_{y} & \mu_{z} \end{array}\right]}^{T}=Q_{b}B_{5}u+Q_{b}B_{6}w,$$

(37h) $$Q_{cn}=\left[\begin{array}{ccc} c_{11} & c_{12} & c_{13}\\ c_{12} & c_{22} & c_{23}\\ c_{13} & c_{23} & c_{33} \end{array}\right],$$

(37i) $$Q_{cs}=\left[\begin{array}{ccc} c_{44} & 0 & 0\\ 0 & c_{55} & 0\\ 0 & 0 & c_{66} \end{array}\right],$$

(37j) $$Q_{b}=\left[\begin{array}{ccc} b_{11} & 0 & 0\\ 0 & b_{22} & 0\\ 0 & 0 & b_{33} \end{array}\right],$$

(37k) $$B_{1}=\left[\begin{array}{cc} \partial_{x} & 0\\ 0 & \partial_{y}\\ 0 & 0 \end{array}\right],$$

(37l) $$B_{2}=\left[\begin{array}{c} 0\\ 0\\ \partial z \end{array}\right],$$

(37m) $$B_{3}=\left[\begin{array}{cc} 0 & \partial z\\ \partial z & 0\\ \partial_{y} & \partial_{x} \end{array}\right],$$

(37n) $$B_{4}=\left[\begin{array}{c} \partial_{y}\\ \partial_{x}\\ 0 \end{array}\right],$$

(37o) $$B_{5}=\left(1/4\right)\left[\begin{array}{cc} -\left(\partial_{yy}+\partial_{zz}\right) & \partial_{xy}\\ \partial_{xy} & -\left(\partial_{xx}+\partial_{zz}\right)\\ \partial_{xz} & \partial_{yz} \end{array}\right],$$

(37p) $$B_{6}=\left(1/4\right)\left[\begin{array}{c} \partial_{xz}\\ \partial_{yz}\\ \left(\partial_{xx}+\partial_{yy}\right) \end{array}\right],$$

where $i=1,2,\ldots ,\;n_{d}$, and $m=1,2,\ldots ,\;n_{l}$.

#### 3.2.2. Galerkin Weak Formulation

Employing Hamilton’s principle and utilizing Equations (34)–(36), we can obtain the Galerkin weak formulation of 3D CCST, which is expressed as follows:

(38) $$\begin{array}{l} \iint_{A}\int_{-h/2}^{h/2}\left\{{\left(\delta u\right)}^{T}\right.\left[{\left({Β}_{1}\right)}^{T}Q_{cn}{Β}_{1}+{\left({Β}_{3}\right)}^{T}Q_{cs}{Β}_{3}-2{\left({Β}_{5}\right)}^{T}Q_{b}{Β}_{5}\right]u\\ \;\;\;+{\left(\delta u\right)}^{T}\left[{\left({Β}_{1}\right)}^{T}Q_{cn}{Β}_{2}\right.+{\left({Β}_{3}\right)}^{T}Q_{cs}{Β}_{4}\left.-2{\left({Β}_{5}\right)}^{T}Q_{b}{Β}_{6}\right]\;u_{z}\\ \;\;\;+\delta u_{z}^{T}\left[{\left({Β}_{2}\right)}^{T}Q_{cn}{Β}_{1}+{\left({Β}_{4}\right)}^{T}Q_{cs}{Β}_{3}-2{\left({Β}_{6}\right)}^{T}Q_{b}{Β}_{5}\right]\;u\\ \;\;\;\left.+\delta u_{z}^{T}\left[{\left({Β}_{2}\right)}^{T}Q_{cn}{Β}_{1}+{\left({Β}_{4}\right)}^{T}Q_{cs}{Β}_{3}-2{\left({Β}_{6}\right)}^{T}Q_{b}{Β}_{5}\right]\;u_{z}\right\}dxdydz\\ \;\;\;-\omega^{2}\iint_{A}\int_{-h/2}^{h/2}\left\{{\left(\delta u\right)}^{T}\rho u+\delta u_{z}^{T}\rho u_{z}\right\}dxdydz+\iint_{A^{+}}\bar{q}\delta u_{z}^{+}dxdy\\ \;\;\;-{\bar{N}}_{x}\iint_{A}\left[\delta u_{z}^{T}{\left(B_{7}\right)}^{T}B_{7}u_{z}\right]dxdy-{\bar{N}}_{y}\iint_{A}\left[\delta u_{z}^{T}{\left(B_{8}\right)}^{T}B_{8}u_{z}\right]dxdy=0, \end{array}$$

where $B_{7}=\left[\partial_{x}\right]$ and $B_{8}=\left[\partial_{y}\right]$.

By incorporating specific kinematic models into Equation (38), Wu and Hsu [68] developed the Lagrangian *C*^0^ and Hermitian *C^n^* FLM in 2022 for investigating the static and dynamic behaviors of FG microplates. They indicated that the differences between the formulations of the CCST and MCST are minor, and most of the different terms fall within the extensional stiffness matrix. After a comparison study, they found that the results of deformations and in-plane stresses obtained using the CCST- and MCST-based FLMs are nearly identical in the static bending analysis. In contrast, the transverse stress solutions differ from one another. In the free vibration analysis, it was found that the results of the lowest natural frequency for the microplate’s flexural modes, obtained using the CCST, are nearly the same as those obtained using the MCST. In contrast, those for the extensional modes using the CCST- and MCST-based FLMs are different from each other. Within the framework of the CSGT-based weak formulation, Wu and Chang [46] developed an LPGM method in 2025 for analyzing the 3D static and dynamic behaviors of an FG microplate under simply supported boundary conditions. In their article, Wu and Chang examined the impact of the dilatational strain gradient, deviatoric strain gradient, and couple-stress tensors on the static and dynamic behaviors of the microplate. The CCST-based FLM and CSGT-based LPGM methods mentioned above were shown to converge rapidly and closely agree with the relevant 3D solutions documented in the literature. The applications of the numerical methods discussed above to various mechanical behaviors of microscale plates and shells [46,68,69,70,71,72,73,74] are listed in Table 3 in chronological order from oldest to most recent.

## 4. Various 2D Shear Deformation Theories

### 4.1. Unified Size-Dependent SDTs

In a unified 2D formulation, the displacements of a microplate, according to various microplate theories, can be written as follows:

(39) $$u_{x}\left(x,y,z,t\right)=u\left(x,y,t\right)-z\frac{\partial w\left(x,y,t\right)}{\partial x}+f\left(z\right)\phi_{x}\left(x,y,t\right),$$

(40) $$u_{y}\left(x,y,z,t\right)=v\left(x,y,t\right)-z\frac{\partial w\left(x,y,t\right)}{\partial y}+f\left(z\right)\phi_{y}\left(x,y,t\right),$$

(41) $$u_{z}\left(x,y,z,t\right)=w\left(x,y,t\right),$$

where u, v, and w denote the microplate’s mid-plane displacements in the *x*, *y*, and *z* directions, respectively. $\phi_{x}\;\mathrm{and}\;\phi_{y}$ are the mid-plane shear rotations of the microplate in the *x*–*z* and *y*–*z* planes, respectively. $f\left(z\right)$ represents a specific function of *z* characterizing the through-thickness distribution of the transverse shear deformations. The displacement fields of various nanoplate theories can be obtained by assigning $f\left(z\right)$ as follows:

(42) $$\mathrm{Classical}\;\mathrm{plate}\;\mathrm{theory}\;(\mathrm{CPT}):\;f\left(z\right)\;=\;0;$$

(43) $$\mathrm{First}\text{-}\mathrm{order}\;\mathrm{shear}\;\mathrm{deformation}\;\mathrm{theory}\;(\mathrm{FOSDT}):\;f\left(z\right)=z;$$

(44) $$\mathrm{Refined}\;\mathrm{shear}\;\mathrm{deformation}\;\mathrm{theory}\;(\mathrm{RSDT}):\;f\left(z\right)=z-\left[\left(4z^{3}\right)/\left(3h^{2}\right)\right];$$

(45) $$\mathrm{Sinusoidal}\;\mathrm{shear}\;\mathrm{deformation}\;\mathrm{theory}\;(\mathrm{SSDT}):\;f\left(z\right)=\left(h/\pi \right)\sin\left(\pi z/h\right);$$

(46) $$\mathrm{Exponential}\;\mathrm{shear}\;\mathrm{deformation}\;\mathrm{theory}\;(\mathrm{ESDT}):\;f\left(z\right)=ze^{-\left(2z^{2}/h^{2}\right)};$$

(47) $$\mathrm{Hyperbolic}\;\mathrm{shear}\;\mathrm{deformation}\;\mathrm{theory}\;(\mathrm{HSDT}):\;f\left(z\right)=z\cosh\left(1/2\right)-h\sinh\left(z/h\right).$$

Incorporating the specific kinematic model expressed in Equations (39)–(41) into the MCST and CCST, Lou et al. [75] and Wu and Hu [76] developed a unified size-dependent SDT for analyzing the mechanical behaviors of elastic isotropic microplates in 2015 and 2021, respectively. Various MCST- and CCST-based SDTs can be reproduced by utilizing their specific function *f*(*z*). Subsequently, several unified size-dependent SDTs [77,78,79,80,81] for analyzing microscale plates and shells made of advanced materials have also been presented and are listed in Table 4 in chronological order, from oldest to most recent.

### 4.2. Size-Dependent Advanced and Refined SDTs

The advanced and refined SDTs, based on the MCST, CCST, MSGT, and CSGT mentioned above, have also been employed to analyze the various mechanical behaviors of microscale plates and shells. The relevant articles are categorized by their use of analytical and numerical methods and are described below.

#### 4.2.1. Variational Analytical Methods

Some variational analytical methods, including the Navier, Nevy, Ritz, and Galerkin methods, have been used to obtain results from various mechanical behavior analyses of EG, FG, sigmoid FG, FG-CNTRC, and FG-GPLRC microscale plates and shells, based on the advanced and refined SDTs mentioned above. These analyses include bending, free vibration, buckling, nonlinear bending, force vibration, nonlinear vibration, post-buckling, and dynamic instability behaviors. The effects of several primary factors on the mechanical behaviors of EG, FG, sigmoid FG, FG-CNTRC, and FG-GPLRC microscale plates and shells have also been examined and discussed. These factors include thickness-stretching, rotation, porosity, imperfection, thermal environment, hygrothermal environment, foundation, sandwich structure, piezoelectricity, and magnetostrictive effects. Partially relevant articles based on the MCST/CCST are listed in Table 5 and Table 6, respectively, for microscale plates [82,83,84,85,86,87,88,89,90,91,92,93,94,95,96,97,98,99,100,101,102,103,104,105,106,107,108,109,110,111,112,113,114,115,116] and shells [117,118,119,120,121,122,123,124,125,126,127,128,129,130,131,132,133,134,135,136,137,138,139,140,141,142,143,144,145], while those based on the MSGT/CSGT are listed in Table 7 and Table 8 for microscale plates [146,147,148,149,150,151,152,153,154,155,156,157,158,159,160,161,162,163,164,165,166,167,168] and shells [126,169,170,171,172,173,174,175,176,177,178,179,180,181]. Finally, within the MSGT framework, Ashoori and Mahmoodi [182] derived the Euler–Lagrange equations and possible boundary conditions of an FG microshell in general curvilinear coordinates. Their results were obtained using a tensor notation approach, which is a general and applicable method for a wide range of problems.

#### 4.2.2. Numerical Methods

The articles referenced in Section 4.2.1 analyze the mechanical behaviors of microscale plates and shells using various variational analytical methods. These methods are limited to examining microstructures under simply-supported boundary conditions and with regular boundary edges, such as rectangular plates, circular annular plates, cylindrical shells, doubly curved shells, and conical shells. To expand the application range of the CCST/MCST and CSGT/MSGT, several numerical methods have been developed for studying the mechanical behaviors of microscale plates and shells, including the differential quadrature (DQ) method, the finite element method (FEM), and the meshless method, which are described below.

##### DQ Method

In the DQ method, each primary variable and its derivatives are expressed as a combination of the nodal function value and an undetermined coefficient, as follows:

(48) $$F\left(x\right)=\sum_{l=1}^{n_{p}}A_{ij}F\left(x_{j}\right),$$

(49) $$\mathrm{and}\;d^{r}F\left(x\right)/dx^{r}=\sum_{l=1}^{n_{p}}A_{ij}^{\left(r\right)}F\left(x_{j}\right),$$

where *n_p_* represents the total number of the sampling nodes; and $A_{ij}$ and $A_{ij}^{\left(r\right)}$ represent the relevant undetermined coefficients for the function *F*(*x*) and its *r*-order derivatives, respectively.

The undetermined coefficients $A_{ij}$ and $A_{ij}^{\left(r\right)}$, given in Equations (48) and (49), can be obtained using a set of Lagrange polynomials and are expressed as follows:

(50) $$A_{ik}^{\left(r\right)}=r\left[A_{ik}^{\left(1\right)}A_{ii}^{\left(r-1\right)}-A_{ik}^{\left(r-1\right)}/\left(x_{i}-x_{k}\right)\right]\;\;\mathrm{when}\;i\neq j,$$

(51) $$\mathrm{and}\;A_{kk}^{\left(r\right)}=-\sum_{\begin{array}{l} i=1,\\ i\neq k \end{array}}^{n_{p}}A_{ik}^{\left(r\right)}\;\;\mathrm{when}\;i=j.$$

The derivations for $A_{ij}^{\left(r\right)}$ and their applications can be found in Du et al. [183], Bert and Malik [184], and Wu and Lee [185]. Partially relevant articles [141,186,187,188,189,190,191,192,193,194,195,196,197] based on the MCST/CCST and MSGT/CSGT, covering various mechanical behaviors of microscale plates and shells using the DQ method, are listed in Table 9.

##### Finite Element Method

When utilizing an FEM, the physical domain of a structure to be analyzed must be divided into multiple elements, where each primary variable is expressed as a combination of the nodal function value and an undetermined coefficient, as follows:

(52) $$F\left(x\right)=\sum_{j=1}^{n_{d}}N_{j}\left(\xi \right)F\left(x_{j}\right),$$

where $N_{j}$ (*j* = 1, 2, …, *n_d_*) represent the shape function for the reference node, which is determined using the Lagrange polynomials as the basis functions and satisfies the nodal interpolation properties; *x* and ξ represent the global and natural coordinates, respectively, and the relationship between them is expressed as $x=\sum_{j=1}^{n_{d}}N_{j}\left(\xi \right)x_{j}$ for an isoparametric FEM; and *n_d_* is the total number of nodes of each element.

The *r*th-order derivative of each variable is expressed as follows:

(53) $$d^{r}F\left(x\right)/dx^{r}=\sum_{j=1}^{n_{d}}\left(d^{r}N_{j}/dx^{r}\right)F\left(x_{j}\right),$$

where *n_p_* represents the total number of sampling nodes; and $A_{ij}$ and $A_{ij}^{\left(r\right)}$ represent the relevant undetermined coefficients for the function $F\left(x\right)$ and its *r*-order derivatives, respectively.

Partially relevant articles [177,184,198,199,200,201,202,203,204,205,206,207,208,209,210,211,212,213,214] based on the MCST/CCST and MSGT/CSGT, covering various mechanical behaviors of microscale plates and shells using the FEM, are listed in Table 10.

##### Meshless Method

Unlike the FEM, a pre-divided mesh is necessary before analysis. The meshless method randomly selects specific nodes within the physical domain, and the approximation function of each variable at the reference node is determined using several neighboring nodes and the least squares method, resulting in a more efficient and less time-consuming process. In the meshless method, each variable is expressed as follows:

(54) $$F\left(x\right)=\sum_{j=1}^{n_{d}}\phi_{j}\left(\xi \right)A\left(x_{j}\right),$$

where $\phi_{j}\left(\xi \right)$ represents the approximation or interpolation function for the reference node, depending on whether the Kronecker delta properties are satisfied or not. Additionally, some basis function sets have been used to determine the approximation function, including the reproducing kernel approximation/interpolation function, the multi-quadric basis function, the moving Kriging interpolation function, and the B-spline basis function.

Partially relevant articles [115,215,216,217,218,219,220,221,222,223,224,225] based on the MCST/CCST and MSGT/CSGT for various mechanical behaviors of microscale plates and shells using the meshless method are listed in Table 11.

## 5. Illustrative Examples

### 5.1. Static Bending Problems

#### 5.1.1. Comparison Studies

Based on the CCST/MCST, we analyze the static bending problem of an FG microplate under simply supported boundary conditions subjected to sinusoidally distributed loads on its top surface, which is expressed as $\bar{q}\left(x,\;y\right)=q_{0}\sin\left(\pi x/L_{x}\right)\;\sin\left(\pi y/L_{y}\right)$. The constituent materials of the microplate are aluminum (Al), a metal, and alumina (Al_2_O_3_), a ceramic. Their material properties are assumed to follow the power-law distributions of the volume fractions of each component through the microplate’s thickness. The effective material properties are expressed in Equation (3), and the ceramic and the metal materials’ volume fractions are provided as follows:

(55a) $$\Gamma_{cer}\left(z\right)={\left[\left(1/2\right)+\left(z/h\right)\right]}^{\kappa_{p}}$$

(55b) $$\mathrm{and}\;\Gamma_{met}\left(z\right)=1-\Gamma_{cer}\left(z\right),$$

where the subscripts *met* and *cer* represent the metal and ceramic materials, respectively. The ceramic and metal materials are fully enriched at the top and bottom surfaces of the microplate, respectively.

The material properties of the metal and ceramic materials are provided as follows [102]:

For the metal material,

(56a) $$E_{met}=7\times 10^{10}\;N/m^{2},$$

(56b) $$\upsilon_{met}=0.3,$$

(56c) $$\rho_{met}=2702\;\mathrm{kg}/m^{3}.$$

For the ceramic material,

(57a) $$E_{cer}=3.8\times 10^{11}\;N/m^{2},$$

(57b) $$\upsilon_{cer}=0.3,$$

(57c) $$\rho_{cer}=3800\;\mathrm{kg}/m^{3}.$$

For comparison, a set of dimensionless stresses and deflections is defined as follows:

(58a) $$\bar{w}=u_{z}\left[10E_{cer}h^{3}/\left(q_{0}L_{x}^{4}\right)\right],$$

(58b) $${\bar{\sigma }}_{ij}=\sigma_{ij}h/\left(q_{0}L_{x}\right).$$

Table 12 compares the results of the microplate’s central deflection and in-plane stress parameters obtained using the 3D CCST-based FEM [68], the Hermitian *C*^2^ DRK meshless method [63], and various 2D MCST-/CCST-based plate theories [76,102], where $L_{x}=L_{y};$ *L_x_*/*h* = 5, 10, and 20; $\kappa_{p}=0,\;1,\;\mathrm{and}\;10;$ $\hat{l}/h=0,\;0.2,\;0.4,0.5,\;0.8,\;\mathrm{and}\;1$. As shown in Table 12, the results from the 3D CCST-based FLM [69] and meshless method [62] are nearly identical. Similarly, the results from the CCST/MCST-based advanced and refined SDTs are the same. For the case of the relative errors between the solutions of the microplate’s central deflection obtained using the 3D FLM and the CCST-based RSDT, they are 0.14% and 1.03%, respectively, when the *l*/*h* ratio is 0 and 0.8, with *L_x_*/*h* = 20. However, these two relative errors increase to 0.65% and 5.3% for the moderately thick microplate (*L_x_*/*h* = 10) and to 2.26% and 12.1% for the thick microplate (*L_x_*/*h* = 5). This indicates that the impact of the material length-scale parameter on the thick microplate’s deflection is more significant than that on the thin microplate’s deflection due to the effects of 3D couple stress, transverse shear deformation, and transverse normal deformation. Additionally, Wu and Hsu [68] noted that the differences between the weak formulations of the 3D CCST and MCST are very minor. Since all the different terms fall within the extensional stiffness matrix, the displacements and in-plane stresses obtained from the CCST and MCST are nearly identical.

#### 5.1.2. Parametric Studies

Based on the CSGT, we perform a parametric study to examine how certain primary factors affect the microplate’s central deflection, including the impact of material length-scale parameters, the inhomogeneity index, and the length-to-thickness ratio. Table 13 and Table 14 compare the results of the microplate’s central deflection and stresses obtained with the CSGT-based LPGM method [46]. The relevant material and geometric parameters are provided as $\kappa_{p}=1,\;5,\;\mathrm{and}\;10;$ *l_i_*/*h* = 0 and 0.5 (*i* = 0, 1, and 2); *L_x_*/*h* = 10 in Table 13, as well as $\kappa_{p}=3;$ *l_i_*/*h* = 0 and 0.5 (*i* = 0, 1, and 2); *L_x_*/*h* = 5, 10, and 20 in Table 14.

As shown in Table 13, any material length-scale parameter increases the overall stiffness of the microplate, leading to a decrease in the microplate’s central deflections and in-plane stresses. For a moderately thick microplate with an *L_x_*/*h* value of 10 and $\kappa_{p}=1$, the microplate’s central deflections decrease by 19.5%, 41.7%, and 55.4% of their original values for the cases of $l_{0}/h=0.5$ and $l_{1}/h=l_{2}/h=0,$ $l_{1}/h=0.5$ and $l_{0}/h=l_{2}/h=0,$ and $l_{2}/h=0.5$ and $l_{0}/h=l_{1}/h=0,$ respectively. The results show that increasing the inhomogeneity index decreases the microplate’s overall stiffness because the volume fraction of the softer metal material rises, leading to greater deflections and stresses.

As shown in Table 14, for a microplate with the value of $\kappa_{p}$ being three and *L_x_*/*h* = 5, the microplate’s central deflections decrease by 16.0%, 31.6%, and 54.7% of their original values for the cases of $l_{0}/h=0.5\;\mathrm{and}\;l_{1}/h=l_{2}/h=0,$ $l_{1}/h=0.5\;\mathrm{and}\;l_{0}/h=l_{2}/h=0,$ and $l_{2}/h=0.5\;\mathrm{and}\;l_{0}/h=l_{1}/h=0,$ respectively. When we convert the results of the dimensionless variables to those of the dimensional variables, it is shown that an increase in the *L_x_*/*h* ratio leads to a decrease in the microplate’s overall stiffness, as the microplate becomes thinner, which increases its deflections and stresses.

**Figure 2:**
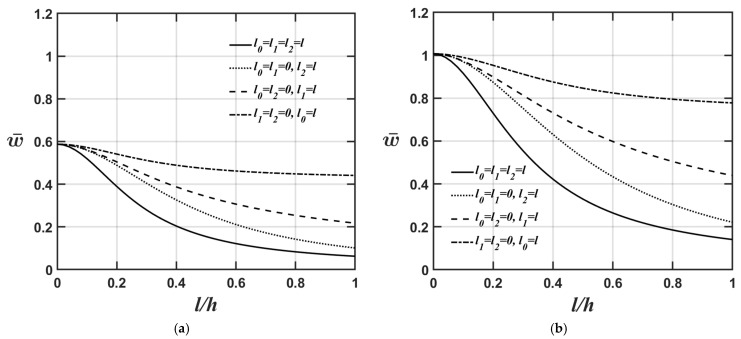
Variations in the microplate’s central deflection with changing the value of the *l*/*h* ratio for different values of the material length-scale parameters, where the relevant geometric and material parameters are $L_{x}=L_{y},\;L_{x}/h=10$ and (**a**) $\kappa_{p}=1$, and (**b**) $\kappa_{p}=10$.

Figure 2a,b show how the microplate’s central deflection varies with different *l*/*h* ratios for four cases: (a) $l_{0}=l\;\mathrm{and}\;l_{1}=l_{2}=0$, considering only the dilatational strain gradient effect; (b) $l_{1}=l\;\mathrm{and}\;l_{0}=l_{2}=0$, considering only the deviatoric strain gradient effect; (c) $l_{2}=l\;\mathrm{and}\;l_{0}=l_{1}=0$, considering only the couple stress effect; and (d) $l_{0}=l_{1}=l_{2}=l$, considering the dilatational strain gradient, deviatoric strain gradient, and couple stress effects. The relevant material and geometric parameters are provided as $L_{x}=L_{y},$ $L_{x}/h=10,$ and $\kappa_{p}=1$ for Figure 2a and $\kappa_{p}=10$ for Figure 2b. As shown in Figure 2a,b again, the higher the $\kappa_{p}$ value, the softer the microplate’s overall stiffness, leading to greater central deflection of the microplate.

**Figure 3:**
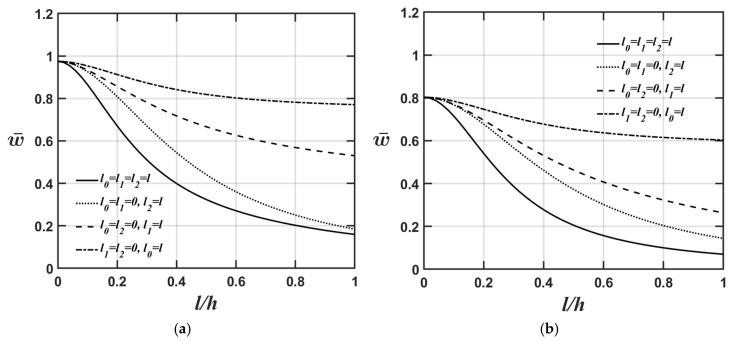
Variations in the microplate’s central deflection with respect to changes in the *l*/*h* ratio for different values of the material length-scale parameters, where the relevant geometric and material parameters are $L_{x}=L_{y},$ $\kappa_{p}=3$, and (**a**) $L_{x}/h=5,$ (**b**) $L_{x}/h=20$.

Figure 3a,b show the variations in the microplate’s central deflection with respect to changes in the *l*/*h* ratio for four different values of the material length-scale parameters. The relevant material and geometric parameters are expressed as $L_{x}=L_{y},$ $\kappa_{p}=3$, and $L_{x}/h=5,$ in Figure 3a, and $L_{x}/h=20,$ in Figure 3b. As shown in Figure 3a,b again, the higher the $L_{x}/h$ value, the softer the microplate’s overall stiffness, leading to greater central deflection of the microplate when we convert the dimensionless microplate’s central deflection to the dimensional microplate’s central deflection. The results shown in Figure 2a,b and Figure 3a,b also demonstrate that the significance of different material length-scale parameters on the microplate’s central deflection is in the following order: couple stress effect > deviatoric strain gradient effect > dilatational strain gradient effect. This is because most of the strain energy contributions from the couple stress effects are associated with the flexural stiffness matrix; however, the contributions from the deviatoric strain gradient effects are associated with the extensional stiffness matrix. Additionally, the strain energy contributions from the dilatational strain gradient effects are smaller, and most of them are associated with the extensional stiffness matrix. This results in the couple stress effects being dominant in the structural behaviors related to the out-of-plane motion and the deviatoric strain gradient effects being dominant in the structural behavior associated with the in-plane motion.

### 5.2. Free Vibration Problems

#### 5.2.1. Comparison Studies

Based on the CCST/MCST, this section examines the free vibration problem of a simply-supported EG microplate, where the material properties are defined as an exponential function through the thickness, as follows [65]:

(59a) $$E\left(\zeta \right)=E_{b}e^{\kappa_{e}\left[\left(1/2\right)+\left(z/h\right)\right]},$$

(59b) $$\rho \left(\zeta \right)=\rho_{b}e^{\kappa_{e}\left[\left(1/2\right)+\left(z/h\right)\right]},$$

(59c) $$\upsilon \left(\zeta \right)=0.3,$$

where *E*, ρ, and υ represent the Young’s modulus, mass density, and Poisson’s ratio; $\kappa_{e}$ represents the inhomogeneity index for the EG microplate, and $\kappa_{e}=\ln{\hat{\kappa }}_{e}=\ln\left(E_{t}/E_{b}\right)=\ln\left(\rho_{t}/\rho_{b}\right)$ and ${\hat{\kappa }}_{e}=\left(E_{t}/E_{b}\right)=\left(\rho_{t}/\rho_{b}\right)$.

For comparison, a non-dimensional frequency parameter is provided as follows [65]:

(60) $$\bar{\omega }=\omega L_{x}^{2}\sqrt{\rho_{b}/E_{b}}/h.$$

Table 15 compares the lowest natural frequency results for the microplate’s flexural modes obtained using the 3D CCST-based Hermitian *C*^1^ DRK meshless method [63], the 3D CCST-based FLM [68], the 3D MCST-based state space method [65], and the CCST-based RSDPT and SSDPT [76]. The relevant material and geometric parameters are expressed as *L_x_* = *L_y_*; *L_x_*/*h* = 5 and 10; l/h=0, 0.2, 0.4, 0.6, 0.8, and 1; $\left(\hat{m},\;\hat{n}\right)=\left(1,\;1\right);$ ${\hat{\kappa }}_{e}=E_{t}/E_{b}=5\;\mathrm{and}\;10$.

As shown in Table 15, the results obtained using the 3D CCST-based Hermitian *C*^1^ DRK meshless method [63], the FLM [68], and the state space analytical method [65] are nearly identical. Similarly, the results obtained using the CCST-based RSDT and SSDT are similar. For a moderately thick microplate with $L_{x}/h$ = 10, the relative errors between the solutions of the microplate’s lowest natural frequency obtained using the 3D MCST and the CCST-based RSDT are 0.05% and 2.9% for the *l*/*h* ratio values of 0 and 1.0, respectively, where ${\hat{\kappa }}_{e}=E_{t}/E_{b}=5$. However, these two relative errors increase to 0.2% and 9.4% for the thick microplate with *L_x_*/*h* = 5. This shows that the impact of the material length-scale parameter on the thick microplate’s lowest natural frequency is more significant than that on the thin microplate’s lowest natural frequency, due to the 3D couple stress effect and the transverse shear and normal deformation effects. Additionally, Wu and Hsu [68] noted that the differences between the weak formulations of the 3D CCST and 3D MCST are very minor. The different terms all fall within the extensional stiffness matrix, leading to nearly identical solutions for the lowest natural frequency of the flexural mode obtained using the CCST and MCST. The only difference occurs in the solutions for the lowest natural frequency of the microplate’s extensional mode from the CCST-based and MCST-based theories.

#### 5.2.2. Parametric Studies

Based on the CSGT, we conduct a parametric study examining the impact of specific primary factors on the lowest natural frequency of the FG microplate, including the effects of material length-scale parameters, the inhomogeneity index, and the length-to-thickness ratio. The constituent materials of the FG microplate are the same as those in Section 5.1; the volume fractions and material properties of these materials are given in Equations (55a,b), (56) and (57), respectively; and the effective material properties of the microplate are described in Equation (3). A dimensionless natural frequency is defined as $\bar{\omega }=\omega L_{x}^{2}\sqrt{\rho_{cer}/E_{cer}}/h.$

Table 16 and Table 17 compare the results of the microplate’s lowest natural frequency obtained using the CSGT-based LPGM method [46], with $\left(\hat{m},\;\hat{n}\right)=(1,\;1)$ and (1, 2). The relevant material and geometric parameters are expressed as $\kappa_{p}=1,\;5,\;\mathrm{and}\;10;$ *l_i_*_/_*h* = 0 and 0.5 (*i* = 0, 1, and 2); and *L_x_*/*h* = 10, as shown in Table 16, as well as $\kappa_{p}=3;$ *l_i_*_/_*h* = 0 and 0.5 (*i* = 0, 1, and 2); and *L_x_*/*h* = 5, 10, and 20, as shown in Table 17. As shown in Table 16, any material length-scale parameters cause the microplate to become stiffer, increasing the lowest natural frequency of its flexural and extensional modes. For a moderately thick microplate with the value *L_x_*/*h* equal to 10 and $\kappa_{p}=1$, the lowest natural frequency of the microplate’s flexural mode increases by 11.5%, 31.0%, and 49.8% of its original value for the cases of $l_{0}/h=0.5\;\mathrm{and}\;l_{1}/h=l_{2}/h=0,$ $l_{1}/h=0.5\;\mathrm{and}\;l_{0}/h=l_{2}/h=0,$ and $l_{2}/h=0.5\;\mathrm{and}\;l_{0}/h=l_{1}/h=0,$ respectively. The relative errors for the lowest natural frequency of the microplate’s extensional mode are 0.2%, 1.3%, and 0%, respectively, for the cases of $l_{0}/h=0.5\;\mathrm{and}\;l_{1}/h=l_{2}/h=0,$ $l_{1}/h=0.5\;\mathrm{and}\;l_{0}/h=l_{2}/h=0,$ and $l_{2}/h=0.5\;\mathrm{and}\;l_{0}/h=l_{1}/h=0$. This indicates that the material length-scale parameter significantly influences the lowest natural frequency of the microplate’s flexural mode, more than its extensional mode. The results also show that increasing the inhomogeneity index decreases the microplate’s overall stiffness because the volume fraction of the softer metal material increases, which in turn reduces the microplate’s lowest natural frequency.

As shown in Table 17, when the results of dimensionless natural frequency are converted to those of the dimensional natural frequency, the results indicate that an increase in the *L_x_*/*h* ratio causes a decrease in the microplate’s overall stiffness because the microplate becomes thinner, which lowers the microplate’s lowest natural frequency.

**Figure 4:**
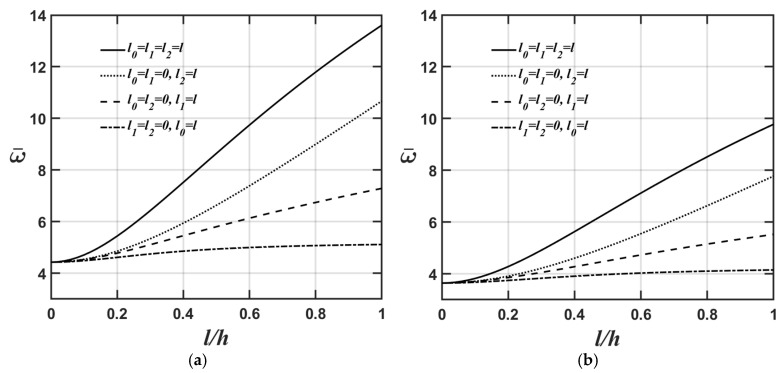
Variations in the lowest natural frequency of the microplate’s flexural mode with respect to changes in the *l*/*h* ratio for different values of the material length-scale parameters, where the relevant geometric and material parameters are $L_{x}=L_{y},$ $L_{x}/h=10,$ and (**a**) $\kappa_{p}=1$, (**b**) $\kappa_{p}=10$.

**Figure 5:**
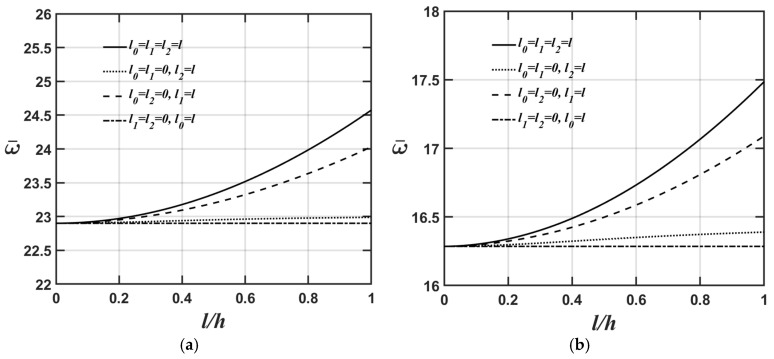
Variations in the lowest natural frequency of the microplate’s extensional mode with respect to changes in the *l*/*h* ratio for different values of the material length-scale parameters, where the relevant geometric and material parameters are $L_{x}=L_{y},$ $L_{x}/h=10,$ and (**a**) $\kappa_{p}=1$, (**b**) $\kappa_{p}=10$.

Figure 4a,b and Figure 5a,b show how the lowest natural frequency of the microplate’s flexural and extensional modes varies with respect to changes in the *l*/*h* ratio for four different values of the material length-scale parameters. The relevant material and geometric parameters are expressed as $L_{x}=L_{y}$; $L_{x}/h=10$; and $\kappa_{p}=1$, as shown in Figure 4a and Figure 5a, and $\kappa_{p}=10$, as shown in Figure 4b and Figure 5b.

As shown in Figure 4a,b and Figure 5a,b, the higher the $\kappa_{p}$ value, the softer the microplate’s overall stiffness, leading to a lower value of the microplate’s lowest natural frequency.

**Figure 6:**
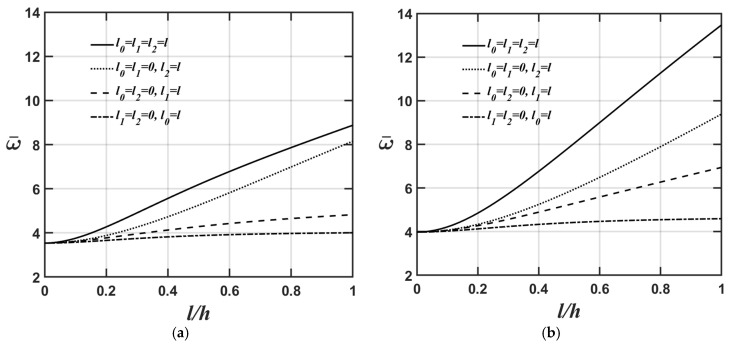
Variations in the lowest natural frequency of the microplate’s flexural mode with respect to changes in the *l*/*h* ratio for different values of the material length-scale parameters, where the relevant geometric and material parameters are $L_{x}=L_{y},$ $\kappa_{p}=3$, and (**a**) $L_{x}/h=5,$ (**b**) $L_{x}/h=20$.

**Figure 7:**
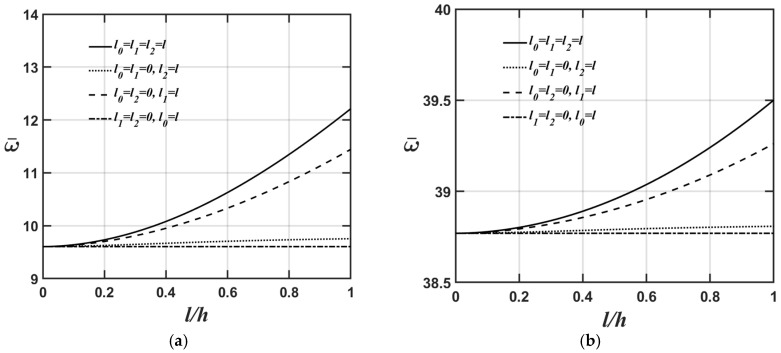
Variations in the lowest natural frequency of the microplate’s extensional mode with respect to changes in the *l*/*h* ratio for different values of the material length-scale parameters, where the relevant geometric and material parameters are $L_{x}=L_{y},$ $\kappa_{p}=3$, and (**a**) $L_{x}/h=5,$ (**b**) $L_{x}/h=20$.

Figure 6a,b and Figure 7a,b display the variations in the lowest natural frequency of the microplate’s flexural and extensional modes, respectively, as the value of the *l*/*h* ratio changes for four different material length-scale parameters. The relevant material and geometric parameters are expressed as $L_{x}=L_{y}$; $\kappa_{p}=3$; and $L_{x}/h=5,$ as shown in Figure 6a and Figure 7a, and $L_{x}/h=20,$ as shown in Figure 6b and Figure 7b.

As shown in Figure 6a,b and Figure 7a,b, the higher the $L_{x}/h$ value, the softer the microplate’s overall stiffness, leading to a lower value of the microplate’s lowest natural frequency when converting the dimensionless lowest natural frequency to the dimensional lowest natural frequency. Furthermore, the results shown in Figure 4a, Figure 5a, Figure 6a and Figure 7a and Figure 4b, Figure 5b, Figure 6b and Figure 7b demonstrate that the impact of material length-scale parameters on the lowest natural frequency of the microplate’s flexural mode is considerably greater than in the microplate’s extensional mode. Additionally, the importance of different material length-scale parameters on the lowest natural frequency of the microplate’s flexural mode is ranked as follows: couple stress effect > deviatoric strain gradient effect > dilatational strain gradient effect; however, for the lowest natural frequency of the microplate’s extensional mode, the order is as follows: deviatoric effect > couple stress effect > dilatational strain gradient effect. As mentioned above, most of the strain energy contributions from the couple stress effects fall within the flexural stiffness matrix; however, those arising from the deviatoric strain gradient effects fall within the extensional stiffness matrix. This results in the couple stress effects being dominant in the lowest natural frequency of the microplate’s flexural mode and the deviatoric strain gradient effects being dominant in the lowest natural frequency of the microplate’s extensional mode.

**Figure 8:**
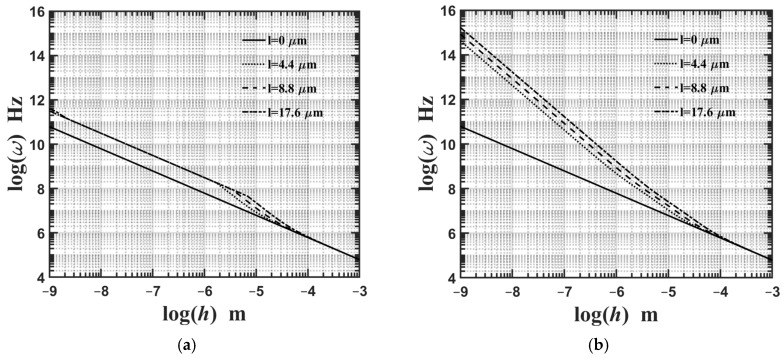
Variations in the lowest natural frequency of the microplate’s flexural mode with respect to changes in its thickness for different values of the material length-scale parameters, where the relevant geometric and material parameters are $L_{x}=L_{y},$ $L_{x}/h=10,$ and $\kappa_{p}=3$ for (**a**) CCST and (**b**) CSGT.

Figure 8a,b show variations in the lowest natural frequency of the microplate’s flexural mode with respect to changes in its thickness based on the CCST and CSGT, respectively. The relevant geometric and material parameters are expressed as $L_{x}=L_{y},$ $L_{x}/h=10,$ and $\kappa_{p}=3$. As shown in Figure 8a,b, the impact of the material length-scale parameter *l*_2_ on the lowest natural frequency of the microplate’s flexural mode becomes saturated when its thickness is less than $1\times 10^{-7}\;m$; conversely, the effects of the dilatational and deviatoric strain gradients consistently influence the microplate’s lowest natural frequency of its flexural mode.

### 5.3. Supplementary Notes

Conducting the comparison and parametric studies, we found that the values of the material length-scale parameters significantly influence the induced deformation results and the lowest natural frequency of the FG microplate. Although the theoretical and numerical methods for analyzing the microplate are well-established, estimating various material length-scale parameters remains a research area with significant potential for development. Additionally, to the best of the authors’ knowledge, the applicable ranges of the structural scale for the CCST/MCST and CSGT/MSGT have not been discussed and recommended. Therefore, a comparison between the results obtained from the experimental test and the theoretical and numerical methods provides another potential research area to be explored.

## 6. Conclusions

This work provides a comprehensive review of studies examining various mechanical behaviors of FG microscale plates and shells based on the MCST/CCST and MSGT/CSGT. We develop and present the strong and weak forms of the 3D CCST and perform a comparative study on the static and dynamic behaviors of a simply supported FG microplate using a range of 3D CCST/MCST-based exact and numerical methods and 2D CCST/MCST-based SDTs. Additionally, based on the 3D CSGT, we conduct a parametric study to investigate how different primary factors affect the deflection, stress, and lowest natural frequency of the FG microplate. Several conclusions from the parametric analysis are summarized as follows:

1. The material length-scale parameters stiffen the FG microplate, resulting in less deflection and an increase in the lowest natural frequency.
2. The higher the value of the inhomogeneity index, the softer the overall stiffness of the FG microplate, leading to a higher value of the microplate’s central deflection and a reduction in its lowest natural frequency.
3. The higher the length-to-thickness ratio, the softer the overall stiffness of the FG microplate, leading to a greater value of the microplate’s central deflection and a reduction in its lowest natural frequency.
4. The importance of the impact of various material length-scale parameters on the FG microplate’s central deflection and the lowest natural frequency of the microplate’s flexural mode is ordered, from greatest to least, as follows: the couple stress effect, the deviatoric strain gradient effect, and finally the dilatational strain gradient effect.
5. The importance of the impact of various material length-scale parameters on the lowest natural frequency of the FG microplate’s extensional mode is arranged, in descending order, as follows: deviatoric strain gradient effect > couple stress effect > dilatational strain gradient effect.
6. The effect of the material length-scale parameters on the lowest natural frequency of the FG microplate’s flexural mode is significantly greater than in its extensional mode.
7. The impact of the material length-scale parameter *l*_2_ on the lowest natural frequency of the microplate’s flexural mode becomes saturated when its thickness is less than $1\times 10^{-7}\;m$; conversely, the effects of the dilatational and deviatoric strain gradients consistently influence the microplate’s lowest natural frequency in its flexural mode.

Since 3D results of deformations, stresses, and natural frequency in an FG microplate are rarely published, the findings in this article can serve as a reference for evaluating the accuracy of the 2D size-dependent advanced and refined SDTs and their relevant numerical methods. Furthermore, the classification of research work provided in the tables will help future scholars gain an overall understanding of related research topics.

## Figures and Tables

**Table 1:** A partial list of articles examining the mechanical behaviors of FG microscale plates and shells using non-CCM theories.

Year	Authors	Non-CCM Theories	Relevant Models	Structures	Mechanical Behaviors
2011	Askes and Aifantis [47]	ENET, Mindlin’s SGT, Aifantis’s SGT.	FEMs	Microplates.	Static bending, Wave propagation.
2015	Hassanpour; Hepple [48]	Micropolar elasticity theory	Experimental tests.	Bone, Polystyrene foams.	Torsion, Bending.
2017	Thai et al. [49]	ENET, MCST.	The Euler–Bernoulli theory, FOSDT, RSDT, HOSDT.	Microbeams, Microplates.	Static bending, Free vibration, Static buckling.
2018	Farajpour et al. [50]	ENET	The Euler–Bernoulli theory, Kirchhoff–Love theory.	Nanorods, Nanobeams, Nanoplates, Nanoshells.	Static bending, Free vibration, Static buckling, Wave propagation.
2019	Ghayesh and Farajpour [51]	ENET, MCST, MSGT.	The Euler–Bernoulli theory, The Kirchhoff–Love theory.	Microbeams, Microplates.	Static bending, Nonlinear free vibration.
2019	Wu and Yu [52]	ENET	The Kirchhoff–Love theory, FOSDT, RSDT, HOSDT.	Nanobeams, Nanoshells, Carbon nanotubes.	Static bending, Free vibration, Static buckling.
2021	Wu and Hu [53]	ENET	The Kirchhoff–Love theory, FOSDT, RSDT, HOSDT.	Nanoplates, Graphene sheets.	Static bending, Free vibration, Static buckling.
2022	Kong [54]	MCST	The Euler–Bernoulli theory, The Kirchhoff–Love theory, FOSDT.	Microbeams, Microplates.	Static bending, Free vibration, Static buckling, Pull-in instability.
2022	Roudbari et al. [55]	ENET, CST, SGT.	The Euler–Bernoulli theory, The Kirchhoff–Love theory, FOSDT.	Microstructures, Nanostructures.	Static bending, Free vibration, Static buckling, Wave propagation.
2022	Nuhu and Safaei [56]	CST, SGT.	The Kirchhoff–Love theory, FOSDT, RSDT, HOSDT.	Microplates, Nanoplates.	Coupled multifield vibration.

CCM: classical continuum mechanics; CST: couple stress theory; ENET: Eringen’s nonlocal elasticity theory; FG: functionally graded; FOSDT: first-order SDT; HOSDT: higher-order shear deformation theory; MCST: modified CST; MSGT: modified SGT; RSDT: refined shear deformation theory; SDT: shear deformation theory; SGT: strain gradient theory.

**Table 2:** A partial list of articles examining the mechanical behaviors of FG microscale plates and shells using the strong formulation of 3D non-CCM theories.

Year	Authors	Non-CCM Theories	Relevant Methods	Structures	Mechanical Behaviors
2015	Salehipour et al. [65]	MCST	State space method	FG micro-/ nano-plates	Free vibration
2018	Salehipour and Shahsavar [67]	MSGT	State space method	FG micro-/ nano-plates	Free vibration
2020	Salehipour et al. [66]	MCST	State space method	FG porous cylindrical micro-/nano-shells	Static bending, Free vibration
2023	Wu and Lyu [64]	CCST	Perturbation method	FG microplates	Free vibration
2024	Wu and Chang [62]	CCST	Meshless DRK point collocation method	FG microplates	Static bending
2025	Wu and Chou [63]	CCST	Meshless DRK point collocation method	FG microplates	Free vibration, Static buckling

CCM: classical continuum mechanics; DRK: differential reproducing kernel; FG: functionally graded; MCST: modified couple stress theory; CCST: consistent couple stress theory; 3D: three-dimensional.

**Table 3:** The articles examining the mechanical behaviors of FG microscale plates and shells using the weak formulations of 3D non-CCM theories.

Year	Authors	Non-CCM Theories	Relevant Methods	Structures	Mechanical Behaviors
2022	Wu and Hsu [68]	CCST	FLM	FG microplates	Static bending, Free vibration
2023	Wu and Lu [69]	CCST	FLM	FG piezoelectric microplates	Free vibration
2023	Wu et al. [70]	CCST	FLM	FG-GPLRC cylindrical shells	Free vibration
2024	Wu et al. [71]	CCST	FLM	FG cylindrical microshells	Dynamic instability
2024	Wu and Hsu [72]	CCST	FLM	FG piezoelectric cylindrical microshells	Static buckling, Free vibration
2023	Wu and Lu [69]	CCST	FLM	FG piezoelectric microplates	Static bending
2025	Wu and Chang [46]	CSGT	LPGM method	FG microplates	Static bending, Free vibration
2025	Wu and Chang [74]	CCST	Finite DRK element method	FG doubly curved microshells	Free vibration

CCST: consistent couple stress theory; CCM: classical continuum mechanics; CSGT: consistent strain gradient theory; DRK: differential reproducing kernel; FG: functionally graded; FLM: finite layer method; LPGM: local Petrov–Galerkin meshless method; 3D: three-dimensional.

**Table 4:** Various unified shear deformation theories for analyzing the mechanical behaviors of microscale plates and shells.

Year	Authors	Non-CCM Theories	Structures	Mechanical Behaviors
2015	Lou et al. [75]	MCST	FG elastic microplates	Static bending, Free vibration, Buckling
2020	Wang et al. [77]	MCST	FG elastic cylindrical microshells	Free vibration
2021	Wu and Hu [76]	CCST	FG elastic microplates	Static bending, Free vibration
2022	Tran et al. [78]	MCST	FG elastic microplates	Stochastic, Vibration, Static buckling
2022	Wu and Lin [79]	CCST	FG piezoelectric microplates	Free vibration
2025	Shaban et al. [80]	CCST	FG microplates	Static bending
2025	Wu and Hsu [81]	CCST	FG magneto–electro–elastic microplates	Free vibration

CCM: classical continuum mechanics; CCST: consistent couple stress theory; FG: functionally graded; MCST: modified couple stress theory.

**Table 5:** A partial list of references on various mechanical behavior analyses of FG microplates using advanced and refined shear deformation theories based on CCST and MCST, implemented using variational analytical methods.

CCST/MCST-Based Size-Dependent Theories	Articles
Classical plate theory	^3, 7^ Tang et al. [82], ^4^ Wang et al. [83], ^6^ Wang et al. [84], ^1, 2, 3^ Thai and Choi [85]
FOSDT	^1, 2, 3^ Thai and Choi [85], ^5, 11, 12^ Simsek and Aydin [86], ^2^ Ke et al. [87], ^1, 2, 3, 11^ Kim et al. [88], ^1, 2, 3^ Yekani and Fallah [89], ^1, 2, 11^ Tounsi et al. [90], ^1, 2, 15^ Jung et al. [91], ^1, 2, 11^ Beitollahi et al. [92], ^1, 2, 4, 11^ Cuong-Le et al. [93], ^1, 16, 15, 17, 18^ Arefi and Kiani [94], ^1, 2, 3, 11, 12^ Van-Hieu et al. [95], ^1, 2, 3, 17, 18^ Wang et al. [96], ^2, 10, 19^ Yin and Fang [97], ^3, 15^ Jung et al. [98]
RSDT	^1, 2, 3, 13, 16^ Trinh et al. [99], ^4, 11^ Tranh et al. [100], ^1, 2^ Lei et al. [101]
TOSDT	^1, 2^ Thai and Kim [102], ^2, 3, 13, 10, 11, 19^ Fang et al. [103], ^1, 11, 19^ Arefi et al. [104], ^1, 2, 3, 11^ Coskun et al. [105]
SSDT	^1, 2, 19^ Afshari and Adab [106], ^6, 20^ Tranh et al. [107], ^3, 16, 17^ Zhang et al. [108], ^1, 2, 3, 14, 15^ Sobhy and Zenkour [109], ^1, 2, 5, 19^ Arefi and Adab [110]
HSDT	^3, 9, 13, 19^ Khorasani et al. [111]
TSNDT	^2, 9^ Mohseni et al. [112], ^1, 9^ Mohseni et al. [113]
4-variable SDT	^2, 3, 9, 19^ Thai et al. [114], ^1, 8, 13, 15, 16, 18, 20^ Radwan and Sobhy [115], ^1, 2, 3^ He et al. [116]

The superscripts 1–20 represent the following: 1: bending, 2: free vibration, 3: buckling, 4: nonlinear bending, 5: force vibration, 6: nonlinear free vibration, 7: post-buckling, 8: dynamic instability, 9: thickness-stretching, 10: rotation, 11: porosity, 12: imperfection, 13: thermal environment, 14: hygro-thermal environment, 15: foundation, 16: sandwich/multilayer, 17: piezoelectricity, 18: magnetostrictive, 19: GPLRC material, and 20: CNTRC material. CCST: consistent couple stress theory; CNTRC: carbon nanotube-reinforced composite; ESDT: exponential SDT; FG: functionally graded; FOSDT: first-order SDT; GPLRC: graphene platelet-reinforced composite; HSDT: hyperbolic SDT; MCST: modified couple stress theory; RSDT: refined SDT; SDT: shear deformation theory; SSDT: sinusoidal SDT; TOSDT: third-order SDT; TSNDT: transverse shear and normal deformation theory.

**Table 6:** A partial list of references on various mechanical behavior analyses of FG microshells using advanced and refined shear deformation theories based on CCST and MCST, implemented using variational analytical methods.

CCST/MCST-Based Size-Dependent Theories	Articles
Classical shell theory	^2, 11^ Mohammadpour et al. [117], ^2, 17^ Razavi et al. [118], ^1, 2, 3^ Tang et al. [119], ^3, 16^ Zeighampour and Shojaeian [120], ^2^ Beni et al. [121], ^4, 6^ Farokhi and Ghayesh [122], ^2, 19^ Wang et al. [123], ^8, 11, 13, 15^ Khuat Duc et al. [124], ^2, 3, 19^ Liu and Wang [125], ^2, 17, 19^ Abbaspour [126].
FOSDT	^2, 11^ Karami et al. [127], ^3^ Lou et al. [128], ^2, 16^ Zeighampour and Shojaeian [129], ^8, 13^ Mirfatah et al. [130], ^3, 13, 17, 19^ Abbaspour and Hosseini [131], ^2, 13, 15^ SafarPour et al. [132], ^2, 17, 19^ Abbaspour and Hosseini [133], ^6^ Veysi et al. [134], ^6, 13, 15^ Sheng and Wang [135], ^2, 11, 13^ Ghadird and SafarPour [136], ^3, 8^ Gholami et al. [137], ^2^ Beni et al. [138], ^1, 2^ Ma et al. [139].
RSDT	^3, 13^ Mehditabar et al. [140], ^2^ Wang et al. [77], ^8^ Sahmani et al. [141].
TOSDT	^1^ Arefi [142].
TSNDT	^2, 9^ Zhang et al. [143], ^2, 9, 17^ Lori Dehsaraji et al. [144], ^3, 9, 13, 17^ Lori Dehsaraji et al. [145].

The superscripts 1–4, 6, 8, 9, 11, 13, 15–17, and 19 represent the following: 1: bending, 2: free vibration, 3: buckling, 4: nonlinear bending, 6: nonlinear free vibration, 8: dynamic instability, 9: thickness-stretching, 11: porosity, 13: thermal environment, 15: foundation, 16: sandwich/multilayer, 17: piezoelectricity, and 19: GPLRC material; CNTRC: carbon nanotube-reinforced composite; FG: functionally graded; FOSDT: first-order SDT; GPLRC: graphene platelet-reinforced composite; MCST: modified couple stress theory; RSDT: refined SDT; SDT: shear deformation theory; TOSDT: third-order SDT; TSNDT: transverse shear and normal deformation theory.

**Table 7:** A partial list of references on various mechanical behavior analyses of FG microplates using advanced and refined shear deformation theories based on CSGT and MSGT, implemented using variational analytical methods.

CSGT/MSGT-Based Size-Dependent Theories	Articles
Classical shell theory	^1^ Li et al. [146], ^1^ Movassagh and Mahmoodi [147], ^1, 2, 3^ Wang et al. [148], ^3, 16^ Hosseini et al. [149], ^3^ Farahmand et al. [150], ^3^ Mohammadi and Mahani [151].
FOSDT	^3, 7, 13^ Ansari et al. [152], ^1, 2, 3^ Ansari et al. [153], ^2^ Markolefas and Fafalis [154], ^1, 2, 16^ Ma et al. [155], ^6^ Gholami and Ansari [156].
RSDT	^1, 2, 3^ Thai et al. [157], ^2, 16, 19^ Thai et al. [158], ^3, 7, 11^ Wang et al. [159], ^2, 11, 17^ Nguyen et al. [160], ^2^ Sahmani and Ansari [161], ^6, 11, 15^ Jain and Kumar [162], ^1, 2, 3, 15^ Zhang et al. [163], ^2, 16, 19^ Hung et al. [164], ^1, 2, 20^ Thai et al. [165].
SSDT	^1, 2, 3^ Akgoz and Civalek [166].
Two-variable SDT	^1, 2, 3^ Farahmand [167].
Four-variable SDT	^1, 2, 3^ Thai et al. [168].

The superscripts 1–3, 6, 7, 11, 13, 15–17, 19, and 20 represent 1: bending, 2: free vibration, 3: buckling, 6: nonlinear free vibration, 7: post-buckling, 11: porosity, 13: thermal environment, 15: foundation, 16: sandwich/multilayer, 17: piezoelectricity, 19: GPLRC material, and 20: CNTRC material. CNTRC: carbon nanotube-reinforced composite; CSGT: consistent strain gradient theory; FG: functionally graded; FOSDT: first-order SDT; GPLRC: graphene platelet-reinforced composite; MSGT: modified strain gradient theory; RSDT: refined SDT; SDT: shear deformation theory; SSDT: sinusoidal SDT.

**Table 8:** A partial list of references on various mechanical behavior analyses of FG microshells using advanced and refined shear deformation theories based on CSGT and MSGT, implemented using variational analytical methods.

CSGT/MSGT-Based Size-Dependent Theories	Articles
Classical shell theory	^2, 3, 19^ Liu and Wang [125], ^4, 6^ Ghayesh and Farokhi [169], ^2, 21^ Zeighampour and Beni [170], ^1, 2^ Qi and Zhou [171].
FOSDT	^7, 8, 20^ Tohidi et al. [172], ^2, 3^ Gholami et al. [173], ^1, 2, 5^ Le et al. [174], ^6, 13, 17^ Movahedfar et al. [175], ^8, 19^ Zhang et al. [176], ^3^ Gholami et al. [177], ^2, 3, 5, 16, 19^ Hajilak et al. [178], ^1, 2, 5^ Le et al. [179].
RSDT	^3^ Krishnan and Ghosh [180].
Four-variable SDT	^2^ Zhang et al. [181].

The superscripts 1–8, 13, 16, 17, and 19–21 represent the following: 1: bending, 2: free vibration, 3: buckling, 4: nonlinear bending, 5: force vibration, 6: nonlinear free vibration, 7: post-buckling, 8: dynamic instability, 13: thermal environment, 16: sandwich/multilayer, 17: piezoelectricity, 19: GPLRC material, 20: CNTRC material, and 21: SWCNT. CNTRC: carbon nanotube-reinforced composite; CSGT: consistent strain gradient theory; FG: functionally graded; FOSDT: first-order SDT; GPLRC: graphene platelet-reinforced composite; MSGT: modified strain gradient theory; RSDT: refined SDT; SDT: shear deformation theory; SWCNT: single-walled carbon nanotube.

**Table 9:** A partial list of references on various mechanical behavior analyses of FG microscale plates and shells using advanced and refined shear deformation theories based on CCST/MCST and CSGT/MSGT, implemented using the differential quadrature method.

CCST/MCST-Based Size-Dependent Theories	Microstructures	Articles
FOSDT	Microplates	^6^ Ansari [186]
	Microshells	^2^ Hosseini-Hashemi [187]
FOSDT	Microplates	^1, 2^ Zhang et al. [188], ^3, 13^ Ansari et al. [189]
	Microshells	^2, 10, 11, 19^ Adab et al. [190]
RSDT	Microplates	^2, 15, 16^ Emdadi et al. [191]
	Microshells	^6^ Yuan et al. [192], ^3, 13^ Mehditabar et al. [140]
TOSDT	Microshells	^6, 18^ Yang et al. [193], ^8, 15, 16, 18^ Fan et al. [194]
Elasticity	Microshells	^1^ Suwankornkij et al. [195], ^2, 3, 11, 17, 19^ Al-Furjan [196], ^1, 2, 13, 17^ Mohammadimehr et al. [197]

The superscripts 1–3, 6, 8, 10, 11, 13, and 15–19 represent the following: 1: bending, 2: free vibration, 3: buckling, 6: nonlinear free vibration, 8: dynamic instability, 10: rotation, 11: porosity, 13: thermal environment, 15: foundation, 16: sandwich/multilayer, 17: piezoelectricity, 18: magnetostrictive, and 19: GPLRC material. CCST: consistent couple stress theory; CNTRC: carbon nanotube-reinforced composite; FG: functionally graded; FOSDT: first-order SDT; GPLRC: graphene platelet-reinforced composite; MCST: modified couple stress theory; RSDT: refined SDT; SDT: shear deformation theory; TOSDT: third-order SDT.

**Table 10:** A partial list of references on various mechanical behavior analyses of FG microscale plates and shells using advanced and refined shear deformation theories based on CCST/MCST and CSGT/MSGT, implemented using the FEM.

CCST/MCST-Based Size-Dependent Theories	Microstructures	Articles
Classical plate/shell theory	Microplates	^1, 2, 5^ Mao et al. [198], ^1, 2, 13^ Wang et al. [199].
	Microshells	^2^ Dehrouyeh-Semnani and Mostafaei [200], ^3^ Soleimani et al. [201], ^1, 2^ Wang et al. [202].
FOSDT	Microplates	^1^ Nguyen et al. [203], ^1, 2, 16, 17^ Korayem and Hefzabad [204], ^1, 2, 3^ Zhang et al. [205].
	Microshells	^3, 11, 19^ Taghizadeh et al. [206].
TOSDT	Microplates	^4, 11, 13^ Genao et al. [207].
TSNDT	Microplates	^2, 11^ Karamanli and Aydogdu [208].
Plane elasticity	Microplates	^1^ Wu et al. [209].
3D elasticity	Microshells	^2, 11, 13^ Wang et al. [210].
Classical shell theory	Microshells	^4^ Thai et al. [211].
FOSDT	Microplates	^1, 2^ Ansari et al. [212].
	Microahells	^1, 2, 5^ Le et al. [174].
RSDT	Microplates	^6^ Zuo et al. [213].
	Microahells	^5^ Li et al. [214].
Four-variable SDT	Microshells	^2^ Zhang et al. [181].

The superscripts 1–6, 11, 13, 16, 17, and 19 represent the following: 1: bending, 2: free vibration, 3: buckling, 4: nonlinear bending, 5: force vibration, 6: nonlinear free vibration, 11: porosity, 13: thermal environment, 16: sandwich/multilayer, 17: piezoelectricity, and 19: GPLRC material. CCST: consistent couple stress theory; CNTRC: carbon nanotube-reinforced composite; FG: functionally graded; FOSDT: first-order SDT; GPLRC: graphene platelet-reinforced composite; MCST: modified couple stress theory; RSDT: refined SDT; SDT: shear deformation theory; TOSDT: third-order SDT.

**Table 11:** A partial list of references on various mechanical behavior analyses of FG microscale plates and shells using advanced and refined shear deformation theories based on CCST/MCST and CSGT/MSGT, implemented using the meshless method.

Basis Functions	CCST/MCST-Based Size-Dependent Theories	Microstructures	Articles
Multi-quadric basis functions	FOSDT	Microplates	^1^ Roque et al. [215], ^1, 2, 5^ Roque and Zur. [216].
Moving Kriging interpolation functions	RSDT	Microplates	^3, 7, 19^ Zhang et al. [217], ^3, 7, 19^ Yang et al. [218].
Moving Kriging interpolation functions	TOSDT	Microshells	^3, 7^ Liu et al. [219].
B-spline basis functions	CPT	Microplates	^1, 2, 3^ Liu et al. [220].
B-spline basis functions	RSDT	Microplates	^4^ Nguyen et al. [221], ^1, 2, 3^ Nguyen et al. [222], ^6, 11^ Fan et al. [223], ^1, 2, 3, 19^ Thai et al. [114].
Moving Kriging interpolation functions	RSDT	Microplates	^1, 2, 3^ Thai et al. [224].
B-spline basis functions	RSDT	Microplates	^2, 3, 13, 17, 18^ Hung et al. [225].

The superscripts 1–5, 7, 11, 13, and 17–19 represent the following: 1: bending, 2: free vibration, 3: buckling, 4: nonlinear bending, 5: force vibration, 7: post-buckling, 11: porosity, 13: thermal environment, 17: piezoelectricity, 18: magnetostrictive, and 19: GPLRC material. CCST: consistent couple stress theory; CNTRC: carbon nanotube-reinforced composite; CPT: classical plate theory; FG: functionally graded; FOSDT: first-order SDT; GPLRC: graphene platelet-reinforced composite; MCST: modified couple stress theory; RSDT: refined SDT; SDT: shear deformation theory; TOSDT: third-order SDT.

**Table 12:** Comparisons of the deflection and stress results induced in a simply-supported FG microplate subjected to the sinusoidally distributed loads obtained using various MCST-/CCST-based microplate theories.

Deflection	$L_{x}/h$	Theories	$\kappa_{p}=0$				$\kappa_{p}=1$				$\kappa_{p}=10$			
			*l*/*h* = 0	*l*/*h* = 0.2	*l*/*h* = 0.4	*l*/*h* = 0.8	*l*/*h* = 0	*l*/*h* = 0.2	*l*/*h* = 0.4	*l*/*h* = 0.8	*l*/*h* = 0	*l*/*h* = 0.2	*l*/*h* = 0.4	*l*/*h* = 0.8
$\bar{w}(L_{x}/2,L_{y}/2,0)$	5	Hermitian *C*^2^ DRK meshless method [62]	0.3357	0.2849	0.1991	0.0953	0.6620	0.5462	0.3652	0.1656	1.2197	1.0421	0.7409	0.3639
		3D CCST-based FLM [68]	0.3357	0.2851	0.1991	0.0953	0.6622	0.5476	0.3653	0.1656	1.2194	1.0394	0.7388	0.3636
		CCST-based SSDT [76]	0.3433	0.2875	0.1934	0.0838	0.6688	0.5468	0.3535	0.1464	1.2276	1.0247	0.6908	0.3052
		CCST-based RSDT [76]	0.3433	0.2875	0.1934	0.0838	0.6688	0.5468	0.3535	0.1464	1.2276	1.0247	0.6908	0.3052
		MCST-based RSDT [102]	0.3433	0.2875	0.1934	0.0838	0.6688	0.5468	0.3535	0.1464	1.2276	1.0247	0.6908	0.3052
		CCST-based CPT [76]	0.2803	0.2399	0.1676	0.0760	0.5623	0.4687	0.3127	0.1341	0.9355	0.8171	0.5922	0.2819
$\bar{w}(L_{x}/2,L_{y}/2,0)$	20	Hermitian *C*^2^ DRK meshless method [62]	0.2838	0.2428	0.1696	0.0773	0.5686	0.4727	0.3161	0.1362	0.9534	0.8337	0.6041	0.2879
		3D CCST-based FLM [68]	0.2838	0.2428	0.1697	0.0773	0.5686	0.4738	0.3161	0.1362	0.9353	0.8315	0.6020	0.2875
		CCST-based SSDT [76]	0.2842	0.2430	0.1693	0.0765	0.5689	0.4737	0.3153	0.1349	0.9538	0.8303	0.5986	0.2834
		CCST-based RSDT [76]	0.2842	0.2430	0.1693	0.0765	0.5689	0.4737	0.3153	0.1349	0.9358	0.8303	0.5986	0.2834
		MCST-based RSDT [102]	0.2842	0.2430	0.1693	0.0765	0.5689	0.4737	0.3153	0.1349	0.9538	0.8303	0.5986	0.2834
		CCST-based CPT [76]	0.2803	0.2399	0.1676	0.0760	0.5623	0.4687	0.3127	0.1341	0.9355	0.8171	0.5922	0.2819
**Deflection and Stress Parameters**	$L_{x}/h$	**Theories**	$\kappa_{p}=0$				$\kappa_{p}=1$				$\kappa_{p}=10$			
			***l*/*h* = 0**	***l*/*h* = 0.2**	***l*/*h* = 0.5**	***l*/*h* = 1**	***l*/*h* = 0**	***l*/*h* = 0.2**	***l*/*h* = 0.5**	***l*/*h* = 1**	***l*/*h* = 0**	***l*/*h* = 0.2**	***l*/*h* = 0.5**	***l*/*h* = 1**
$\bar{w}(L_{x}/2,L_{y}/2,0)$	10	Hermitian *C*^2^ DRK meshless method [62]	0.2943	0.2514	0.1437	0.0583	0.5875	0.4876	0.2618	0.1013	1.0074	0.8790	0.5273	0.2216
		3D CCST-based FLM [68]	0.2942	0.2514	0.1437	0.0583	0.5875	0.4888	0.2617	0.1012	1.0073	0.8734	0.5228	0.2212
		CCST-based SSDT [76]	0.2960	0.2519	0.1414	0.0551	0.5889	0.4884	0.2576	0.0959	1.0089	0.8704	0.5085	0.2060
		CCST-based RSDT [76]	0.2961	0.2520	0.1415	0.0552	0.5890	0.4885	0.2577	0.0959	1.0087	0.8697	0.5079	0.2058
		MCST-based RSDT [102]	0.2960	0.2520	NA	0.0552	0.5890	0.4885	NA	0.0959	1.0087	0.8697	NA	0.2058
		CCST-based CPT [76]	0.2803	0.2399	0.1367	0.0539	0.5623	0.4687	0.2502	0.0939	0.9355	0.8171	0.4909	0.2024
${\bar{\sigma }}_{xx}(L_{x}/2,L_{y}/2,h/2)$	10	Hermitian *C*^2^ DRK meshless method [62]	2.0049	1.7099	0.9700	0.3841	3.0978	2.5638	1.3597	0.5046	5.0662	4.4161	2.6079	1.0235
		3D CCST-based FLM [68]	2.0044	1.7103	0.9700	0.3841	3.0973	2.5701	1.3592	0.5044	5.0633	4.3906	2.5857	1.0205
		CCST-based SSDT [76]	1.9955	1.7002	0.9579	0.3750	3.0870	2.5613	1.3530	0.5044	5.0890	4.4135	2.6258	1.0727
		CCST-based RSDT [76]	1.9943	1.6992	0.9575	0.3750	3.0850	2.5598	1.3524	0.5042	5.0849	4.4103	2.6156	1.0733
		MCST-based SSDT [226]	1.9955	1.6945	0.9528	0.3762	3.0870	2.5541	1.3467	0.5048	5.0890	4.4019	2.6050	1.0737
		CCST-based CPT [76]	1.9758	1.6916	0.9638	0.3799	3.0537	2.5456	1.3588	0.5099	5.0173	4.3824	2.6330	1.0855
${\bar{\sigma }}_{xy}(0,0,-h/3)$	10	Hermitian *C*^2^ DRK meshless method [62]	0.7083	0.6057	0.3437	0.1346	0.6111	0.5067	0.2689	0.0989	0.5926	0.5185	0.3091	0.1239
		3D CCST-based FLM [68]	0.7085	0.6058	0.3437	0.1346	0.6112	0.5081	0.2689	0.0989	0.5927	0.5156	0.3065	0.1237
		CCST-based SSDT [76]	0.7065	0.6022	0.3396	0.1331	0.6110	0.5071	0.2680	0.1000	0.5894	0.5120	0.3049	0.1255
		CCST-based RSDT [76]	0.7067	0.6023	0.3398	0.1332	0.6111	0.5072	0.2681	0.1000	0.5896	0.5123	0.3053	0.1257
		MCST-based SSDT [68]	0.7065	0.6007	0.3392	0.1345	0.6110	0.5061	0.2677	0.1007	0.5894	0.5111	0.3044	0.1262
		CCST-based CPT [76]	0.7093	0.6072	0.3460	0.1364	0.6125	0.5106	0.2726	0.1023	0.5926	0.5176	0.3110	0.1282

CCST: consistent couple stress theory; CPT: classical plate theory; DRK: differential reproducing kernel; FLM: finite layer method; MCST: modified couple stress theory; NA: not available; RSDT: refined shear deformation theory; SSDT: sinusoidal shear deformation theory; 3D: three-dimensional.

**Table 13:** Comparison of results for the deflections and stresses induced in an FG square microplate with fully simple supports, obtained using the CSGT-based LPGM method, where *L_x_/h* = 10 [46].

$\kappa_{p}$	$l_{0}/h$	$l_{1}/h$	$l_{2}/h$	$\bar{w}\left(L_{x}/2,\;L_{y}/2,\;0\right)$	${\bar{\sigma }}_{xx}\left(L_{x}/2,\;L_{y}/2,\;h/2\right)$	${\bar{\sigma }}_{xy}\left(0,\;0,\;-h/3\right)$
1	0	0	0	0.5875	3.0958	0.6112
	0	0	0.5	0.2618	1.3583	0.2689
	0	0.5	0	0.3426	2.3704	0.3437
	0.5	0	0	0.4730	1.6522	0.4860
	0.5	0.5	0.5	0.1546	0.6532	0.1459
5	0	0	0	0.9118	4.2559	0.5765
	0	0	0.5	0.4463	2.0481	0.2810
	0	0.5	0	0.5745	3.4625	0.3554
	0.5	0	0	0.7592	2.4555	0.4729
	0.5	0.5	0.5	0.2741	1.0462	0.1596
10	0	0	0	1.0074	5.0644	0.5927
	0	0	0.5	0.5231	2.5860	0.3065
	0	0.5	0	0.65892	4.1671	0.3793
	0.5	0	0	0.8466	3.0601	0.4907
	0.5	0.5	0.5	0.3294	1.3766	0.1789

**Table 14:** Comparison of results for the deflections and stresses induced in an FG square microplate with fully simple supports, obtained using the CSGT-based LPGM method, where $\kappa_{p}=3$.

*L_x_/h*	$l_{0}/h$	$l_{1}/h$	$l_{2}/h$	$\bar{w}\left(L_{x}/2,\;\;L_{y}/2,\;\;0\right)$	${\bar{\sigma }}_{xx}\left(L_{x}/2,\;\;L_{y}/2,\;\;h/2\right)$	${\bar{\sigma }}_{xy}\left(0,\;\;0,\;\;-h/3\right)$
5	0	0	0	0.9750	2.0507	0.2721
	0	0	0.5	0.4412	0.8691	0.1202
	0	0.5	0	0.6666	1.6566	0.1756
	0.5	0	0	0.8189	1.1251	0.2177
	0.5	0.5	0.5	0.3239	0.4401	0.0724
10	0	0	0	0.8381	3.8898	0.5522
	0	0	0.5	0.3872	1.7662	0.2534
	0	0.5	0	0.5063	3.0844	0.3248
	0.5	0	0	0.6874	2.1485	0.4465
	0.5	0.5	0.5	0.2322	0.8707	0.1400
20	0	0	0	0.8033	7.6772	1.1082
	0	0	0.5	0.3729	3.5477	0.5135
	0	0.5	0	0.4632	6.0591	0.6341
	0.5	0	0	0.6539	4.2395	0.8988
	0.5	0.5	0.5	0.2056	1.7341	0.2765

**Table 15:** Comparisons of the results for the lowest frequency parameter solutions of the flexural modes (i.e., the out-of-plane modes) for a simply supported, EG microplate, obtained using various CCST/MCST-based microplate theories.

$L_{x}/h$	*E_t_*/*E_b_*	Theories	l/h=0	l/h=0.2	l/h=0.4	l/h=0.6	l/h=0.8	l/h=1
5	5	3D DRK meshless point method [63]	5.0168	5.5043	6.7133	8.2707	9.9435	11.6073
		3D CCST-based FLM [68]	5.0168	5.5043	6.7133	8.2707	9.9435	11.6073
		3D MCST [65]	5.0168	5.5043	6.7133	8.2707	9.9435	11.607
		CCST-based RSDPT [76]	5.0088	5.5286	6.8528	8.6156	10.5995	12.7014
		CCST-based SSDPT [76]	5.0089	5.5286	6.8530	8.6160	10.6004	12.7028
5	10	3D DRK meshless point method [63]	4.7524	5.2747	6.5481	8.1611	9.8741	11.5655
		3D CCST-based FLM [68]	4.7524	5.2745	6.5480	8.1611	9.8741	11.5655
		3D MCST [65]	4.7524	5.2745	6.5480	8.1611	9.8741	11.5660
		CCST-based RSDPT [76]	4.7550	5.3058	6.6883	8.5031	10.5283	12.6634
		CCST-based SSDPT [76]	4.7545	5.3049	6.6870	8.5017	10.5270	12.6623
10	5	3D DRK meshless point method [63]	5.4392	5.9427	7.2307	8.9499	10.8718	12.8764
		3D CCST-based FLM [68]	5.4392	5.9424	7.2307	8.9499	10.8717	12.8764
		3D MCST [65]	5.4392	5.9424	7.2307	8.9499	10.872	12.876
		CCST-based RSDPT [76]	5.4365	5.9501	7.2756	9.0624	11.0896	13.2472
		CCST-based SSDPT [76]	5.4364	5.9500	7.2754	9.0623	11.0896	13.2473
10	10	3D DRK meshless point method [63]	5.1295	5.6638	7.0110	8.7827	10.7443	12.7786
		3D CCST-based FLM [68]	5.1295	5.6635	7.0109	8.7827	10.7443	12.7785
		3D MCST [65]	5.1295	5.6635	7.0109	8.7827	10.744	12.779
		CCST-based RSDPT [76]	5.1302	5.6734	7.0553	8.8920	10.9569	13.1430
		CCST-based SSDPT [76]	5.1300	5.6730	7.0547	8.8913	10.9562	13.1422

CCST: consistent couple stress theory; CPT: classical plate theory; DRK: differential reproducing kernel; FLM: finite layer method; MCST: modified couple stress theory; RSDT: refined shear deformation theory; SSDT: sinusoidal shear deformation theory; 3D: three-dimensional.

**Table 16:** The results for the natural frequency of an FG square microplate with fully simple supports obtained using the CSGT-based LPGM method, where *L_x_/h* = 10.

$\kappa_{p}$	$l_{0}/h$	$l_{1}/h$	$l_{2}/h$	$\left(\hat{m},\;\hat{n}\right)=(1,\;1)$		$\left(\hat{m},\;\hat{n}\right)=(1,\;2)$	
				Flexural Mode	Extensional Mode	Flexural Mode	Extensional Mode
1	0	0	0	4.4258	22.9003	10.6265	36.1557
	0	0	0.5	6.6319	22.9513	15.9332	36.3549
	0	0.5	0	5.7995	23.1987	12.9510	37.3279
	0.5	0	0	4.9347	22.9003	11.7962	36.1557
	0.5	0.5	0.5	8.6414	23.3314	19.3672	37.8375
5	0	0	0	3.7716	17.8505	8.9274	28.1231
	0	0	0.5	5.3906	17.8968	12.8578	28.3059
	0	0.5	0	4.7512	18.0916	10.6085	29.0717
	0.5	0	0	4.1375	17.8505	9.7425	28.1231
	0.5	0.5	0.5	6.8896	18.1963	15.3976	29.4726
10	0	0	0	3.6412	16.2854	8.5853	25.6901
	0	0	0.5	5.0532	16.3371	12.0107	25.8937
	0	0.5	0	4.5033	16.4981	10.0661	26.5257
	0.5	0	0	3.9745	16.2854	9.3176	25.6901
	0.5	0.5	0.5	6.3767	16.5995	14.2301	26.9156

**Table 17:** The results for the natural frequency of an FG square microplate with fully simple supports obtained using the CSGT-based LPGM method, where $\kappa_{p}=3$.

*L_x_/h*	$l_{0}/h$	$l_{1}/h$	$l_{2}/h$	$\left(\hat{m},\;\;\hat{n}\right)=(1,\;\;1)$		$\left(\hat{m},\;\;\hat{n}\right)=(1,\;\;2)$	
				Flexural Mode	Extensional Mode	Flexural Mode	Extensional Mode
5	0	0	0	3.5368	9.6049	7.7359	14.9660
	0	0	0.5	5.2572	9.6881	11.7347	15.2897
	0	0.5	0	4.2867	10.1261	8.7027	16.9791
	0.5	0	0	3.8769	9.6049	8.4040	14.9660
	0.5	0.5	0.5	6.1913	10.3311	12.8685	17.7029
10	0	0	0	3.8727	19.3499	9.2260	30.4842
	0	0	0.5	5.6976	19.3918	13.6422	30.6492
	0	0.5	0	4.9825	19.6127	11.1169	31.5187
	0.5	0	0	4.2806	19.3499	10.1498	30.4842
	0.5	0.5	0.5	7.3689	19.7220	16.4938	31.9368
20	0	0	0	3.9780	38.7696	9.8100	61.2448
	0	0	0.5	5.8390	38.7905	14.4156	61.3277
	0	0.5	0	5.2378	38.9012	12.3431	61.7647
	0.5	0	0	4.4101	38.7696	10.8587	61.2448
	0.5	0.5	0.5	7.8645	38.9568	18.7472	61.9827

## Data Availability

No new data were created or analyzed in this study. Data sharing is not applicable to this article.

## Abbreviations/Notations

The following abbreviations and notations are used in this manuscript:

Abbreviations	
CCM	Classical continuum mechanics
CCST	Consistent couple stress theory
CNT	Carbon nanotube
CNTRC	Carbon nanotubes-reinforced composite
CPT	Classical plate theory
CSGT	Consistent strain gradient theory
CST	Couple stress theory
DQ	Differential quadrature
DRK	Differential reproducing kernel
EG	Exponentially graded
ENET	Eringen’s nonlocal elasticity theory
ESDT	Exponential shear deformation theory
FEM	Finite element method
FG	Functionally graded
FLM	Finite layer method
FOSDT	First-order shear deformation theory
GPL	Graphene platelet
GPLRC	Graphene platelet-reinforced composite
HOSDT	Higher-order shear deformation theory
HSDT	Hyperbolic shear deformation theory
LPGM	Local Petrov–Galerkin meshless
MCST	Modified couple stress theory
MSGT	Modified strain gradient theory
RSDT	Refined shear deformation theory
SDT	Shear deformation theory
SGT	Strain gradient theory
SSDT	Sinusoidal shear deformation theory
SWCNT	Single-walled carbon nanotubes
TOSDT	Third-order shear deformation theory
TSNDT	Transverse shear and normal deformation theory
UD	Uniformly distributed
2D	Two-dimensional
3D	Three-dimensional
Notations	
$A_{ij},\;A_{ij}^{\left(r\right)}$	The relevant undetermined coefficients for unknowns and its *r*-th derivatives for the DQ method
$B\left(\sigma_{ij},\mu_{k}\right)$	The complementary strain energy
$B_{k}$	The relevant matrices in the Galerkin weak formulation
$c_{ij}$	The elastic coefficients
$E_{eff}$	The effective Young’s modulus of a typical material
${\left(E_{ll}\right)}_{k}$	Young’s modulus in the *l*-direction of a typical material k
$E_{L}$	The longitudinal modulus of a typical material
$E_{T}$	The transverse modulus of a typical material
*f*(*z*)	A specific function of z characterizing the through-thickness direction of the transverse shear deformations
${\left(G_{ij}\right)}_{k}$	Shear modulus related to the *ij*-plane of a typical material k
*h*	The thickness of a microplate
$l,\;\hat{l}$	The material length-scale parameters for the MCST and CCST
$l_{0},\;l_{1},\;l_{2}$	The material length-scale parameters related to the effects of the dilatational and deviatoric strain gradients and couple stress for the CSGT and MSGT
*L*	The Lagrange functional
$L_{x}$	The length of a microplate
$L_{y}$	The width of a microplate
$m_{ij}^{b}$	A general material property on the bottom surface of a microplate
$m_{ij}^{t}$	A general material property on the top surface of a microplate
$n_{x},\;n_{y}$	The components of the unit normal vectors of the edges of a microplate
$N_{j}\left(\xi \right)$	The shape functions for the reference point *l* for the finite element method
${\bar{N}}_{ij}^{0}$	The applied resultant forces
$q_{0}$	The magnitude of the applied load
$Q_{b},\;Q_{cn},\;Q_{cs}$	The relevant stiffness matrices
*T*	The kinetic energy
*u*	The mid-plane displacement in the x-direction of a microplate
$u_{i}$	The displacement tensor
*v*	The mid-plane displacement in the y-direction of a microplate
*w*	The mid-plane displacement in the *z*-direction of a microplate
$\bar{w}$	The dimensionless deflection of a microplate
*W*	The work performed
$W_{k}\left(z\right)$	The weight fraction of a typical material k
$W_{k}^{*}$	The weight index of a typical material k
$\Gamma_{k}\left(z\right)$	The volume fraction of a typical material k
$\Gamma_{k}^{*}$	The volume fraction index of a typical material k
δ	The variational operator
$\varepsilon_{ij}$	The strain tensor
$\varepsilon_{ij}^{nl}$	The von Kármán second-order strains
$\eta_{L}$$,\;\eta_{T}$	The parameters describing the geometric dimensions of GPLs
$\kappa_{e}$	The inhomogeneity index for an EG microplate
$\kappa_{p}$	The inhomogeneity index for the power-law FG microplate
$\kappa_{s}$	The inhomogeneity index for the sigmoid FG microplate
${\hat{\kappa }}_{e}$	The material-property ratio between the top and bottom surfaces of a microplate
$\kappa_{ij}$	The skew-symmetric part of the curvature tensor
$\lambda_{i}$	The CNT efficiency parameters
$\mu_{ij}$	The couple-stress tensor
$\xi_{L}$$,\;\xi_{T}$	The parameters characterizing the geometrical dimensions of GPLs
$\Pi_{R}$	Reissner’s strain energy
$\rho_{k}$	The mass density of a typical material k
$\sigma_{\left(ij\right)}$	The symmetric part of the force-stress tensor
$\sigma_{\left[ij\right]}$	The skew-symmetric part of the force-stress tensor
${\bar{\sigma }}_{ij}$	The dimensionless stress tensor
$\upsilon_{ij}$	Poisson’s ratio for transverse strain in the *j*-direction when stressed in the *i*-direction
$\phi_{j}\left(\xi \right)$	The interpolation function of a reference node *j* for the meshless method
$\phi_{x},\;\phi_{y}$	The mid-plane shear rotation of a microplate
$\chi_{ij}$	The symmetric part of the curvature tensor
$\omega ,\;\bar{\omega }$	Natural frequency and its dimensionless form
$\Omega_{ij}$	The rotation tensor

## References

[1] Koizumi M., Jhon B.W. (1992). Recent progress of functionally graded materials in Japan. Proceedings of the 16th Annual Conference on Composites and Advanced Ceramic Materials: Ceramic Engineering and Science Proceedings.

[2] Koizumi M. (1997). FGM activities in Japan. Compos. Part B.

[3] Van Doan D., Van Minh P., Van Ke T., Nhung N.T.C., Van Thom D. (2025). An overview of functionally graded materials: From civil applications to defense and aerospace industries. J. Vib. Eng. Technol..

[4] Shen H.S. (2009). Functionally Graded Materials: Nonlinear Analysis of Plates and Shells.

[5] Nikbakht S., Kamarian S., Shakeri M. (2019). A review on optimization of composite structures Part II: Functionally graded materials. Compos. Struct..

[6] Ding S., Wu C.P. (2018). Optimization of material composition to minimize the thermal stresses induced in FGM plates with temperature-dependent material properties. Int. J. Mech. Mater. Des..

[7] Wu C.P., Li K.M. (2021). Multi-objective optimization of functionally graded beams using a genetic algorithm with non-dominated sorting. J. Compos. Sci..

[8] Zhang N., Khan T., Guo H., Shi S., Zhong W., Zhang W. (2019). Functionally graded materials: An overview of stability, buckling, and free vibration analysis. Adv. Mater. Sci. Eng..

[9] Ghatage P.S., Kar V.R., Sudhagar P.E. (2020). On the numerical modelling and analysis of multi-directional functionally graded composite structures: A review. Compos. Struct..

[10] Jha D.K., Kant T., Singh R.K. (2013). A critical review of recent research on functionally graded plates. Compos. Struct..

[11] Punera D., Kant T. (2019). A critical review of stress and vibration analyses of functionally graded shell structures. Compos. Struct..

[12] Liew K.M., Zhao X., Ferreira A.J.M. (2011). A review of meshless methods for laminated and functionally graded plates and shells. Compos. Struct..

[13] Thai H.T., Kim S.E. (2015). A review of theories for the modeling and analysis of functionally graded plates and shells. Compos. Struct..

[14] Chen D., Gao K., Zhang L. (2023). Functionally graded porous structures: Analyses, performances, and applications—A review. Thin-Walled Struct..

[15] Wu C.P., Liu Y.C. (2016). A review of semi-analytical numerical methods for laminated composite and multilayered functionally graded elastic/piezoelectric plates and shells. Compos. Struct..

[16] Wu C.P., Chiu K.H., Wang Y.M. (2008). A review on the three-dimensional analytical approaches of multilayered and functionally graded piezoelectric plates and shells. CMC—Comput. Mater. Contin..

[17] Li Z., He Y., Lei J., Han S., Guo S., Liu D. (2019). Experimental investigation on size-dependent higher-mode vibration of cantilever microbeams. Microsyst. Technol..

[18] Lei J., He Y., Guo S., Li Z., Liu D. (2016). Size-dependent vibration of nickel cantilever microbeams: Experment and gradient elasticity. Aip Adv..

[19] Ince R., Yalcin E., Arslan A. (2007). Size-dependent response of dowel action in R.C. members. Eng. Struct..

[20] Chang T.H., Cheng G., Li C., Zhu Y. (2016). On the size-dependent elasticity of penta-twinned silver nanowires. Extrem. Mech. Lett..

[21] Pharr G.M., Herbert E.G., Gao Y. (2010). The indentation size effect: A critical examination of experimental observations and mechanistic interpretations. Annu. Rev. Mater..

[22] Fleck N.A., Muller G.M., Ashby M.F., Hutchinson J.W. (1994). Strain gradient plasticity: Theory and experiment. Acta Metall. Mater..

[23] Lam D.C.C., Yang F., Chong A.C.M., Wang J., Tong P. (2003). Experiments and theory in strain gradient elasticity. J. Mech. Phys. Solids.

[24] McFarland A.W., Colton J.S. (2005). Role of material microstructure in plate stiffness with relevance to microcantilever sensors. J. Micromech. Microeng..

[25] Eringen A.C., Eringen A.C. (1999). Theory of micropolar elasticity. Microcontinuum Field Theories.

[26] Fares M.E., Salem M.G., Atta D., Elmarghany M.K. (2023). Mixed variational principle for micropolar elasticity and an accurate two-dimensional plate model. Eur. J. Mech. A/Solids.

[27] Athanasiadis A.E.F., Budzik M.K., Fernando D., Dias M.A. (2024). On micropolar elastic foundations. Eur. J. Mech. A/Solids.

[28] Eringen A.C., Edelen D.G.B. (1972). On nonlocal elasticity. Int. J. Eng. Sci..

[29] Eringen A.C. (1974). Theory of nonlocal thermoelasticity. Int. J. Eng. Sci..

[30] Eringen A.C. (1983). Theories of nonlocal plasticity. Int. J. Eng. Sci..

[31] Shaat M., Ghavanloo E., Fazelzadeh S.A. (2020). Review on nonlocal continuum mechanics: Physics, material applicability, and mathematics. Mech. Mater..

[32] Mindlin R.D., Eshel N.N. (1968). On first strain-gradient theories in linear elasticity. Int. J. Solids Struct..

[33] Polyzos D., Fotiadis D.I. (2012). Derivation of Mindlin’s first and second strain gradient elastic theory via simple lattice and continuum models. Int. J. Solids Struct..

[34] Zhao J., Pedroso D. (2008). Strain gradient theory in orthogonal curvilinear coordinates. Int. J. Solids Struct..

[35] Ferrari M., Granik V.T., Imam A., Ferrari M., Granik V.T., Imam A., Nadeau J.C. (1997). Introduction to doublet mechanics. Advanced in Doublet Mechanics: 45.

[36] Mon K., Ferrari M., Ferrari M., Granik V.T., Imam A., Nadeau J.C. (1997). Doublet thermomechanics. Advanced in Doublet Mechanics: 45.

[37] Granik V.T., Ferrari M., Granik V.T., Imam A., Nadeau J.C. (1997). Comparison with other theories. Advanced in Doublet Mechanics: 45.

[38] Mindlin R.D., Tierstem H.F. (1962). Effects of couple-stresses in linear elasticity. Arch. Ration. Mech. Anal..

[39] Toupin R.A. (1964). Theories of elasticity with couple-stress. Arch. Ration. Mech. Anal..

[40] Koiter W.T. (1964). Couple stresses in the theory of elasticity I and II. Phil. Trans. Roy. Soc. Lond. B.

[41] Yang F., Chong A.C.M., Lam D.C.C., Tong P. (2002). Couple stress-based strain gradient theory for elasticity. Int. J. Solids Struct..

[42] Hadjesfandiari A.R., Dargush G.F. (2011). Couple stress theory for solids. Int. J. Solids Struct..

[43] Hadjesfandiari A.R., Dargush G.F. (2013). Fundamental solutions for isotropic size-dependent couple stress elasticity. Int. J. Solids Struct..

[44] Hadjesfandiari A.R. (2014). Size-dependent thermoelasticity. Latin Amer. J. Solids Struct..

[45] Hadjesfandiari A.R. (2013). Size-dependent piezoelectricity. Int. J. Solids Struct..

[46] Wu C.P., Chang T.Y. (2025). A comparative study of consistent couple stress and strain gradient theories on the mechanical behaviors of functionally gradient microplates using the local Petrov-Galerkin meshless method. Thin-Walled Struct..

[47] Askes H., Aifantis E.C. (2011). Gradient elasticity in statics and dynamics: An overview of formulations, length scale identification procedures, finite element implementations and new results. Int. J. Solids Struct..

[48] Hassanpour S., Heppler G.R. (2015). Micropolar elasticity theory: A survey of linear isotropic equations, representative notations, and experimental investigations. Math. Mech. Solids.

[49] Thai H.T., Vo T.P., Nguyen T.K., Kim S.E. (2017). A review of continuum mechanics models for size-dependent analysis of beams and plates. Compos. Struct..

[50] Farajpour A., Ghayesh M.H., Farokhi H. (2018). A review on the mechanics of nanostructures. Int. J. Eng. Sci..

[51] Ghayesh M.H., Farajpour A. (2019). A review on the mechanics of functionally graded nanoscale and microscale structures. Int. J. Eng. Sci..

[52] Wu C.P., Yu J.J. (2019). A review of mechanical analysis of rectangular nanobeams and single-, double-, and multi-walled carbon nanotubes using Eringen’s nonlocal elasticity theory. Arch. Appl. Mech..

[53] Wu C.P., Hu H.X. (2021). A review of dynamic analyses of single-, and multi-layered graphene sheets/nanoplates using various nonlocal continuum mechanics-based plate theories. Acta Mech..

[54] Kong S. (2022). A review on the size-dependent models of micro-beam and micro-plate based on the modified couple stress theory. Arch. Computat. Methods Eng..

[55] Roudbari M.A., Jorshari T.D., Lu C., Ansari R., Kouzani A.Z., Amabili M. (2022). A review of size-dependent continuum mechanics models for micro- and nano-structures. Thin-Walled Struct..

[56] Nuhu A.A., Safaei B. (2022). A comprehensive review on the vibration analyses of small-scaled plate-based structures by utilizing the nonclassical continuum elasticity theories. Thin-Walled Struct..

[57] Shen H.S., Xiang Y. (2012). Nonlinear vibration of nanotube-reinforced composite cylindrical shells in thermal environments. Comput. Methods Appl. Mech. Eng..

[58] Zhao S., Zhao Z., Yang Z., Ke L., Kitipornchai S., Yang J. (2020). Functionally graded graphene reinforced composite structures: A review. Eng. Struct..

[59] Song M., Kitipornchai S., Yang J. (2017). Free and forced vibrations of functionally graded polymer composite plates reinforced with graphene platelets. Compos. Struct..

[60] Reddy J.N. (2017). Energy and Variational Methods in Applied Mechanics.

[61] Wang Y.M., Chen S.M., Wu C.P. (2010). A meshless collocation method based on the differential reproducing kernel interpolation. Comput. Mech..

[62] Wu C.P., Chang R.S. (2024). A Hermitian C^2^ differential reproducing kernel interpolation meshless method for the 3D microstructure-dependent static flexural analysis of simply supported and functionally graded microplates. CMES-Comput. Methods Eng. Sci..

[63] Wu C.P., Chou Y.Q. (2025). A size-dependent meshless differential reproducing kernel point method for static buckling and free vibration analyses of functionally graded microplates subjected to bi-axial compression. Acta Mech..

[64] Wu C.P., Lyu Y.S. (2023). An asymptotic consistent couple stress theory for the three-dimensional free vibration analysis of functionally graded microplates resting on an elastic medium. Math. Methods Appl. Sci..

[65] Salehipour H., Nahvi H., Shahidi A.R. (2015). Exact closed-form free vibration analysis for functionally graded micro/nano plates based on modified couple stress and three-dimensional elasticity theories. Compos. Struct..

[66] Salehipour H., Shahgholian-Ghahfarokhi D., Shahsavar A., Civalek O., Edalati M. (2020). Static deflection and free vibration analysis of functionally graded and porous cylindrical micro/nano shells based on the three-dimensional elasticity and modified couple stress theories. Mech. Based Des. Struct. Mach..

[67] Salehipour H., Shahsavar A. (2018). A three-dimensional elasticity model for free vibration analysis of functionally graded micro/nano plates: Modified strain gradient theory. Compos. Struct..

[68] Wu C.P., Hsu C.H. (2022). A three-dimensional weak formulation for stress, deformation, and free vibration analyses of functionally graded microscale plates based on the consistent couple stress theory. Compos. Struct..

[69] Wu C.P., Lu Y.A. (2023). A Hermite-family C^1^ finite layer method for the three-dimensional free vibration analysis of exponentially graded piezoelectric microplates based on the consistent couple stress theory. Int. J. Struct. Stab. Dyn..

[70] Wu C.P., Tan T.F., Hsu H.T. (2023). A size-dependent finite element method for the 3D free vibration analysis of functionally graded graphene platelets-reinforced composite cylindrical microshells based on the consistent couple stress theory. Materials.

[71] Wu C.P., Wu M.L., Hsu H.T. (2024). 3D size-dependent dynamic instability analysis of FG cylindrical microshells subjected to combinations of periodic axial compression and external pressure using a Hermitian C2 finite layer method based on the consistent couple stress theory. Materials.

[72] Wu C.P., Hsu H.T. (2024). A Hermitian C^n^ finite cylindrical layer method for 3D size-dependent buckling and free vibration analyses of simply supported FG piezoelectric cylindrical sandwich microshells subjected to axial compression and electric voltages. ZAMM-J. Appl. Math. Mech..

[73] Wu C.P., Lu Y.S. (2024). 3D static bending analysis of functionally graded piezoelectric microplates resting on an elastic medium subjected to electro-mechanical loads using a size-dependent Hermitian C^2^ finite layer method based on the consistent couple stress theory. Mech. Bas. Des. Struct. Mach..

[74] Wu C.P., Chang R.S. (2025). Semi-analytical differential reproducing kernel element method for the size-dependent free vibration characteristics analysis of functionally graded doubly curved microscale shells. Int. J. Struct. Stab. Dyn..

[75] Lou J., He L., Du J. (2015). A unified higher order plate theory for functionally graded microplates based on the modified couple stress theory. Compos. Struct..

[76] Wu C.P., Hu H.X. (2021). A unified size-dependent plate theory for static bending and free vibration analyses of micro- and nano-scale plates based on the consistent couple stress theory. Mech. Mater..

[77] Wang Y., Xie K., Fu T., Zhang W. (2020). A unified modified couple stress model for size-dependent free vibrations of FG cylindrical microshells based on high-order shear deformation theory. Eur. Phys. J. Plus.

[78] Tran V.T., Nguyen T.K., Nguyen P.T.T., Vo T.P. (2022). Stochastic vibration and buckling analysis of functionally graded microplates with a unified higher-order shear deformation theory. Thin-Walled Struct..

[79] Wu C.P., Lin E.L. (2022). Free vibration analysis of porous functionally graded piezoelectric microplates resting on an elastic medium subjected to electric voltages. Arch. Mech..

[80] Shaban M., Minaeii S., Kalhori H. (2025). Size-dependent flexural analysis of thick microplates using consistent couple stress theory. J. Compos. Sci..

[81] Wu C.P., Hsu C.D. (2025). A unified size-dependent theory for analyzing the free vibration behavior of an FG microplate under fully simply supported conditions and magneto-electro-thermo-mechanical loads considering couple stress and thickness stretching effects. J. Compos. Sci..

[82] Tang F., Dong F., Guo Y., Shi S., Jiang J., Liu S. (2022). Size-dependent buckling and post-buckling analysis of the functionally graded thin plate Al-Cu material based on a modified couple stress theory. Nanomaterials.

[83] Wang Y.G., Lin W.H., Zhou C.L. (2014). Nonlinear bending of size-dependent circular microplates based on the modified couple stress theory. Arch. Appl. Mech..

[84] Wang Y.G., Lin W.H., Liu N. (2013). Large amplitude free vibration of size-dependent circular microplates based on the modified couple stress theory. Int. J. Mech. Sci..

[85] Thai H.T., Choi D.H. (2013). Size-dependent functionally graded Kirchhoff and Mindlin plate models based on a modified couple stress theory. Compos. Struct..

[86] Simsek M., Aydin M. (2017). Size-dependent forced vibration of an imperfect functionally graded (FG) microplate with porosities subjected to a moving load using the modified couple stress theory. Compos. Struct..

[87] Ke L.L., Wang Y.W., Yang J., Kitipornchai S. (2012). Free vibration of size-dependent Mindlin microplates based on the modified couple stress theory. J. Sound Vib..

[88] Kim J., Zur K.K., Reddy J.N. (2019). Bending, free vibration, and buckling of modified couple stress-based functionally graded porous microplates. Compos. Struct..

[89] Yekani S.M.A., Fallah F. (2020). A Levy solution for bending, buckling, and vibration of Mindlin microplates with a modified couple stress theory. SN Appl. Sci..

[90] Tounsi A., Kaci A., Tounsi A., Al-Osta M.A., Yaylaci M., Mohamed S.M.Y., Althobaiti S., Selim M.M. (2025). Quasi-3D plate theory for size-dependent static and free vibration analysis of FG microplate with porosities based on a modified couple stress theory. Mech. Adv. Mater. Struct..

[91] Jung W.Y., Park W.T., Han S.C. (2014). Bending and vibration analysis of S-FGM microplates embedded in Pasternak elastic medium using the modified couple stress theory. Int. J. Mech. Sci..

[92] Beitollahi A., Bazargan-Lari Y., Janghorban M. (2024). On the variable length scale parameter in functionally graded non-porous and porous microplate/nanoplate. Mech. Adv. Mater. Struct..

[93] Cuong-Le T., Hoang-Le M., Ferreira A.J.M., Wahab M.A. (2022). Small size-effect isogeometric analysis for linear and nonlinear responses of porous metal foam microplate. Compos. Struct..

[94] Arefi M., Kiani M. (2018). Magneto-electro-mechanical bending analysis of three-layered exponentially graded microplate with piezomagnetic face-sheets resting on Pasternak’s foundation via MCST. Mech. Adv. Mater. Struct..

[95] Van Hieu D., Hoa N.T., Chan D.Q. (2023). Size-dependent mechanical analysis of imperfect FG Mindlin microplate with porosities resting on elastic foundation through the modified couple stress theory. Iran J. Sci. Technol. Trans. Mech. Eng..

[96] Wang S., Hong J., Yin S., Zhang G. (2024). Isogeometric analysis of magneto-electro-elastic functionally graded Mindlin microplates. Thin-Walled Struct..

[97] Yin B., Fang J. (2023). Modified couple stress-based free vibration and dynamic responses of rotating FG multilayer composite microplates reinforced with graphene platelets. Arch. Appl. Mech..

[98] Jung W.Y., Han S.C., Park W.T. (2014). A modified couple stress theory for buckling analysis of S-FGM nanoplates embedded in Pasternak elastic medium. Compos. Part B.

[99] Trinh L.C., Vo T.P., Thai H.T., Mantari J.L. (2017). Size-dependent behavior of functionally graded sandwich microplates under mechanical and thermal loads. Compos. Part B.

[100] Tranh C.L., Nguyen T.N., Vu T.H., Khatir S., Wahab M.A. (2022). A geometrically nonlinear size-dependent hypothesis for porous functionally graded microplate. Eng. Comput..

[101] Lei J., He Y., Zhang B., Liu D., Shen L., Guo S. (2015). A size-dependent FG microplate model incorporating higher-order shear and normal deformation effects based on a modified couple stress theory. Int. J. Mech. Sci..

[102] Thai H.T., Kim S.E. (2013). A size-dependent functionally graded Reddy plate model based on a modified couple stress theory. Compos. Part B.

[103] Fang J., Yin B., Li L., Zhang D. (2023). Thermal buckling and vibration analysis of rotating porous FG GNPs-reinforced Reddy microplates. Aerosp. Sci. Technol..

[104] Arefi M., Firouzeh S., Bidgoli M.R.E., Civalek O. (2020). Analysis of porous microplates reinforced with FG-GNPs based on Reddy plate theory. Compos. Struct..

[105] Coskun S., Kim J., Toutanji H. (2019). Bending, free vibration, and buckling analysis of functionally graded porous microplates using a general third-order plate theory. J. Compos. Sci..

[106] Afshari H., Adab N. (2020). Size-dependent buckling and vibration analyses of GNP reinforced microplates based on the quasi-3D sinusoidal shear deformation theory. Mech. Based Des. Struct. Mach..

[107] Thanh C.L., Tran L.V., Vu-Huu T., Nguyen-Xuan H., Abdel-Wahab M. (2019). Size-dependent nonlinear analysis and damping responses of FG-CNTRC micro-plates. Comput. Methods Appl. Mech. Eng..

[108] Zhang C., Eyvazian A., Alkhedher M., Alwetaishi M., Ahammad N.A. (2022). Modified couple stress theory application to analyze mechanical buckling behavior of three-layer rectangular microplates with honeycomb core and piezoelectric face sheets. Compos. Struct..

[109] Sobhy M., Zenkour A.M. (2019). A comprehensive study on the size-dependent hygrothermal analysis of exponentially graded microplates on elastic foundations. Mech. Adv. Mater. Struct..

[110] Arefi M., Adab N. (2021). Coupled stress based formulation for static and dynamic analyses of a higher-order shear and normal deformable FG-GPL reinforced microplates. Waves Random Complex Media.

[111] Khorasani M., Soleimani-Javid Z., Arshid E., Lampani L., Civalek O. (2021). Thermal-elastic buckling of honeycomb micro plates integrated with FG-GNPs reinforced epoxy skins with stretching effect. Compos. Struct..

[112] Mohseni E., Saidi A.R., Mohammadi M. (2019). Vibration analysis of thick functionally graded microplates using HOSNDPT and modified couple stress theory. Iran J. Sci. Technol. Trans. Mech. Eng..

[113] Mohseni E., Saidi A.R., Mohammadi M. (2017). Bending-stretching analysis of thick functionally graded microplates using higher-order shear and normal deformable plate theory. Mech. Adv. Mater. Struct..

[114] Thai C.H., Ferreira A.J.M., Tran T.D., Phung-Van P. (2020). A size-dependent quasi-3D isogeometric model for functionally graded graphene platelet-reinforced composite microplates based on the modified couple stress theory. Compos. Struct..

[115] Radwan A.F., Sobhy M. (2020). Transient instability analysis of viscoelastic sandwich CNTs-reinforced microplates exposed 2D magnetic field and hygrothermal conditions. Compos. Struct..

[116] He L., Lou J., Zhang E., Wang Y., Bai Y. (2015). A size-dependent four variable refined plate model for functionally graded microplates based on modified couple stress theory. Compos. Struct..

[117] Mohammadpour A., Mehrabadi S.J., Yousefi P., Mohseni-Monfared H. (2022). Free vibration analysis of functionally graded porous elliptical microshells using modified couple stress theory. Waves Random Complex Media.

[118] Razavi H., Babadi A.F., Beni Y.T. (2017). Free vibration analysis of functionally graded piezoelectric cylindrical nanoshell based on consistent couple stress theory. Compos. Struct..

[119] Tang F., He S., Shi S., Xue S., Dong F., Liu S. (2022). Analysis of size-dependent linear static bending, buckling, and free vibration based on a modified couple stress theory. Materials.

[120] Zeighampour H., Shojaeian M. (2017). Buckling analysis of functionally graded sandwich cylindrical micro/nanoshells based on the couple stress theory. J. Sandw. Struct. Mater..

[121] Beni T., Mehralian F., Zeighampour H. (2016). The modified couple stress functionally graded cylindrical thin shell formulation. Mech. Adv. Mater. Struct..

[122] Farokhi H., Ghayesh M.H. (2018). Nonlinear mechanical behavior of microshells. Int. J. Eng. Sci..

[123] Wang Y.Q., Liu Y.F., Zu J.W. (2019). Size-dependent vibration of circular cylindrical polymeric microshells reinforced with graphene platelets. Int. J. Appl. Mech..

[124] Khuat Duc D., Nguyen Tuan L., Dao Nhu M., Hong N.T., Van Ke T., Minh P.V. (2024). A novel isogeometric model for dynamic buckling analysis of doubly curved two-directional functionally graded porous shallow microshells in thermal environments via variable length-scale parameters. Mech. Based Des. Struct. Mach..

[125] Liu Y., Wang Y. (2019). Size-dependent free vibration and buckling of three-dimensional graphene foam microshells based on modified couple stress theory. Materials.

[126] Abbaspour F. (2021). Free vibration analysis of simply-supported graphene platelets reinforced laminated piezoelectric cylindrical microshells. Int. J. Comput. Methods Eng. Sci. Mech..

[127] Karami B., Ghayesh M.H., Hussain S., Amabili M. (2024). On the size-dependent vibrations of doubly curved porous shear deformable FGM microshells. Int. J. Mech. Syst. Dyn..

[128] Lou J., He L., Wu H., Du J. (2016). Pre-buckling and buckling analyses of functionally graded microshells under axial and radial loads based on the modified couple stress theory. Compos. Struct..

[129] Zeighampour H., Shojaeian M. (2017). Size-dependent vibration of sandwich cylindrical nanoshells with functionally graded material based on the couple stress theory. J. Braz. Soc. Mech. Sci. Eng..

[130] Mirfatah S.M., Shahmohammadi M.A., Salehipour H. (2022). Size-dependent dynamic stability of nanocomposite enriched microshell panels in thermal environment using the modified couple stress theory. Eng. Anal. Bound. Elem..

[131] Abbaspour F., Hosseini S. (2023). Thermal buckling of piezoelectric graphene platelets reinforced cylindrical microshells using Navier’s and meshless methods. Mech. Based Des. Struct. Mach..

[132] SafarPour H., Hosseini M., Ghadiri M. (2017). Influence of three-parameter viscoelastic medium on vibration behavior of a cylindrical nonhomogeneous microshell in thermal environment: An exact solution. J. Therm. Stress..

[133] Abbaspour F., Hosseini S. (2022). Free vibration analyses of graphene platelets reinforced laminated piezoelectric cylindrical microshells using the Chebyshev-Ritz formulation. J. Vib. Eng. Technol..

[134] Veysi A., Shabani R., Rezazadeh G. (2017). Nonlinear vibration of micro-doubly curved shallow shells based on the modified couple stress theory. Nonlinear Dyn..

[135] Sheng G.G., Wang X. (2022). Nonlinear resonance responses of size-depenednt functionally graded cylindrical microshells with thermal effect and elastic medium. Eng. Comput..

[136] Ghadiri M., SafarPour H. (2016). Free vibration analysis of size-dependent functionally graded porous cylindrical microshells in thermal environment. J. Therm. Stress..

[137] Gholami R., Ansari R., Darvizeh A., Sahmani S. (2015). Axial buckling and dynamic stability of functionally graded microshells based on the modified couple stress theory. Int. J. Struct. Stab. Dyn..

[138] Beni Y.T., Mehralian F., Razavi H. (2015). Free vibration analysis of size-dependent shear deformable functionally graded cylindrical shell on the basis of modified couple stress theory. Compos. Struct..

[139] Ma H.M., Gao X.L., Reddy J.N. (2011). A non-classical Mindlin plate model based on a modified couple stress theory. Acta Mech..

[140] Mehditabar A., Ansari Sadrabadi S., Walker J. (2021). Thermal buckling analysis of a functionally graded microshell based on higher-order shear deformation and modified couple stress theories. Mech. Based. Des. Struct. Mach..

[141] Sahmani S., Ansari R., Gholami R., Darvizeh A. (2013). Dynamic stability analysis of functionally graded higher-order shear deformable microshells based on the modified couple stress theory. Compos. Part B.

[142] Arefi M. (2024). MCST bending formulation of a cylindrical microshell based on TSDT. Earthq. Struct..

[143] Zhang M., Jiang X., Arefi M. (2023). Dynamic formulation of a sandwich microshell considering modified couple stress and thickness-stretching. Eur. Phys. J. Plus.

[144] Lori Dehsaraji M., Arefi M., Loghman A. (2021). Size dependent free vibration analysis of functionally graded piezoelectric micro/nano shell based on modified couple stress theory with considering thickness stretching. Def. Technol..

[145] Lori Dehsaraji M., Loghman A., Arefi M. (2020). Three-dimensional thermo-electro-mechanical buckling analysis of functionally graded piezoelectric micro/nano-shells based on modified couple stress theory considering thickness stretching effect. Mech. Adv. Mater. Struct..

[146] Li A., Zhou S., Zhou S., Wang B. (2014). A size-dependent model for bi-layered Kirchhoff micro-plate based on strain gradient elasticity theory. Compos. Struct..

[147] Movassagh A.A., Mahmoodi M.J. (2013). A microscale modeling of Kirchhoff plate based on modified strain-gradient elasticity theory. Eur. J. Mech. A/Solids.

[148] Wang B., Zhou S., Zhao J., Chen X. (2011). A size-dependent Kirchhoff micro-plate model based on strain gradient elasticity theory. Eur. J. Mech. A/Solids.

[149] Hosseini M., Bahreman M., Jamalpoor A. (2016). Using the modified strain gradient theory to investigate the size-dependent biaxial buckling analyses of an orthotropic multi-microplate system. Acta Mech..

[150] Farahmand H., Naseralav S.S., Iranmanesh A., Mohammadi M. (2016). Navier solution for buckling analysis of size-dependent functionally graded microplates. Lat. Am. J. Solids Struct..

[151] Mohammadi M., Mahani M.F. (2015). An analytical solution for buckling analysis of size-dependent rectangular micro-plates according to the modified strain gradient and couple stress theories. Acta Mech..

[152] Ansari R., Shojaei M.F., Mohammsdi V., Gholami R., Rouhi H. (2013). Size-dependent thermal buckling and postbuckling of functionally graded annular microplates based on the modified strain gradient theory. J. Therm. Stress..

[153] Ansari R., Gholami R., Shojaei M.F., Mohammadi V., Sahmani S. (2015). Bending, buckling and free vibration analysis of size-dependent functionally graded circular/annular microplates based on the modified strain gradient elasticity theory. Eur. J. Mech. A/Solids.

[154] Markolefas S., Fafalis D. (2021). Strain gradient theory based dynamic Mindlin-Reissner and Kirchhoff microplates with microstructural and micro-inertial effects. Dynamics.

[155] Ma B., Chen K.Y., Habibi M., Albaijan I. (2023). Static/dynamic analyses of sandwich microplate based on modified strain gradient theory. Mech. Adv. Mater. Struct..

[156] Gholami R., Ansari R. (2016). A most general strain gradient plate formulation for size-dependent geometrically nonlinear free vibration analysis of functionally graded shear deformable rectangular microplates. Nonlinear Dyn..

[157] Thai S., Thai H.T., Vo T.P., Patel V.I. (2017). Size-dependent behavior of functionally graded microplates based on the modified strain gradient elasticity theory and isogeometric analysis. Comput. Struct..

[158] Thai C.H., Ferreira A.J.M., Phung-Van P. (2019). Size dependent free vibration analysis of multilayer functionally graded GPLRC microplates based on modified strain gradient theory. Compos. Part B.

[159] Wang J., Ma B., Gao J., Liu H., Safaei B., Sahmani S. (2022). Nonlinear stability characteristics of porous graded composite microplates including various microstructural-dependent strain gradient tensors. Int. J. Appl. Mech..

[160] Nguyen L.B., Thai C.H., Duong-Nguyen N., Nguyen-Xuan H. (2022). A size-dependent isogeometric approach for vibration analysis of FG piezoelectric porous microplates using modified strain gradient theory. Eng. Comput..

[161] Sahmani S., Ansari R. (2013). On the free vibration response of functionally graded higher-order shear deformable microplates based on the strain gradient elasticity theory. Compos. Struct..

[162] Jain V., Kumar R. (2023). Geometrically nonlinear dynamic analysis of a damped porous microplate resting on elastic foundations under transverse patch loadings. Mech. Adv. Mater. Struct..

[163] Zhang B., He Y., Liu D., Shen L., Lei J. (2015). An efficient size-dependent plate theory for bending, buckling, and free vibration analyses of functionally graded microplates resting on elastic foundation. Appl. Math. Modell..

[164] Hung P.T., Thai C.H., Phung-Van P. (2024). Isogeometric free vibration of honeycomb sandwich microplates with the graphene nanoplatelets reinforcement face sheets. Eng. Struct..

[165] Thai C.H., Ferreira A.J.M., Rabczuk T., Nguyen-Xuan H. (2018). Size-dependent analysis of FG-CNTRC microplates based on modified strain gradient elasticity theory. Eur. J. Mech. A/Solids.

[166] Akgoz B., Civalek O. (2015). A microstructure-dependent sinusoidal plate model based on the strain gradient elasticity theory. Acta Mech..

[167] Farahmand H. (2021). A variational approach for analytical buckling solution of moderately thick microplate using strain gradient theory incorporating two-variable refined plate theory: A benchmark study. J. Braz. Soc. Mech. Sci. Eng..

[168] Thai C.H., Ferreira A.J.M., Nguyen-Xuan H. (2016). Isogeometric analysis of size-dependent isotropic and sandwich functionally graded microplates based on modified strain gradient elasticity theory. Compos. Struct..

[169] Ghayesh M.H., Farokhi H. (2017). Nonlinear mechanics of doubly curved shallow microshells. Int. J. Eng. Sci..

[170] Zeighampour H., Beni Y.T. (2014). Cylindrical thin-shell model based on modified strain gradient theory. Int. J. Eng. Sci..

[171] Qi L., Zhou S. (2020). A size-dependent spherical microshell model based on strain gradient elasticity theory. Eur. J. Mech. A/Solids.

[172] Tohidi H., Hosseini-Hashemi S.H., Maghsoudpour A. (2017). Nonlinear size-dependent dynamic buckling analysis of embedded micro cylindrical shells reinforced with agglomerated CNYs using strain gradient theory. Microsyst. Technol..

[173] Gholami R., Darvizeh A., Ansari R., Sadeghi F. (2016). Vibration and buckling of first-order shear deformable circular cylindrical micro-/nano-shells based on Mindlin’s strain gradient elasticity theory. Eur. J. Mech. A/Solids.

[174] Le T.M., Vo D., Aung Z.Y., Atroshchenko E., Bui T.Q., Rungamornrat J. (2024). Isogeometric analysis of shear-deformable, in-plane functionally graded microplates by Mindlin’s strain gradient theory. Eng. Comput..

[175] Movahedfar V., Kheirikhah M.M., Mohammadi Y., Ebrahimi F. (2021). Modified strain gradient theory for nonlinear vibration analysis of functionally graded piezoelectric doubly curved microshells. Proc. Instit. Mech. Eng. Sci..

[176] Zhang F., Bai C.Y., Zhang Y., Cao D.Y. (2022). Dynamic stability analysis of functionally graded three-dimensional graphene form cylindrical microshells using interior pressure based on modified strain gradient theory. Eur. Phys. J. Plus.

[177] Gholami R., Darvizeh A., Ansari R., Hosseinzadeh M. (2014). Size-dependent axial buckling analysis of functionally graded circular cylindrical microshells based on the modified strain gradient elasticity theory. Meccanica.

[178] Hajilak Z.E., Pourghader J., Hashmabadi D., Bagh F.S., Habibi M., Safarpour H. (2019). Multilayer GPLRC composite cylindrical nanoshell using modified strain gradient theory. Mech. Based Des. Struct. Mach..

[179] Le T.M., Vo D., Rungamornrat J., Bui T.Q. (2022). Strain-gradient theory for shear deformation free-form microshells: Governing equations of motion and general boundary conditions. Int. J. Solids Struct..

[180] Krishnan N.M.A., Ghosh D. (2017). Buckling analysis of cylindrical thin-shells using strain gradient elasticity theory. Meccanica.

[181] Zhang B., He Y., Liu D., Shen L., Lei J. (2015). Free vibration analysis of four-unknown shear deformable functionally graded cylindrical microshells based on the strain gradient elasticity theory. Compos. Struct..

[182] Ashoori A., Mahmoodi M.J. (2015). The modified version of strain gradient and couple stress theories in general curvilinear coordinates. Eur. J. Mech. A/Solids.

[183] Du H., Lim M.K., Lin R.M. (1994). Application of generalized differential quadrature method to structural problems. Int. J. Numer. Methods Eng..

[184] Bert C.W., Malik M. (1997). Differential quadrature: A powerful new technique for analysis of composite structures. Compos. Struct..

[185] Wu C.P., Lee C.Y. (2001). Differential quadrature solution for the free vibration analysis of laminated conical shells with variable stiffness. Int. J. Mech. Sci..

[186] Ansari R., Shojaei M.F., Mohammadi V., Gholami R., Darabi M.A. (2014). Nonlinear vibrations of functionally graded Mindlin microplates based on the modified couple stress theory. Compos. Struct..

[187] Hosseini-Hashemi S., Sharifpour F., Ilkhani M.R. (2016). On the free vibrations of size-dependent closed micro/nano-spherical shell based on the modified couple stress theory. Int. J. Mech. Sci..

[188] Zhang B., Li H., Kong L., Zhang X., Feng Z. (2020). Strain gradient differential quadrature finite element for moderately thick microplates. Int. J. Numer. Methods Eng..

[189] Ansari R., Gholami R., Faghih Shojaei M., Mohammadi V., Darabi M.A. (2013). Thermal buckling analysis of a Mindlin rectangular FGM microplate based on the strain gradient theory. J. Therm. Stress..

[190] Adab N., Arefi M., Amabili M. (2022). A comprehensive vibration analysis of rotating truncated sandwich conical microshells including porous core and GPL-reinforced face-sheets. Compos. Struct..

[191] Emdadi M., Mohammadimehr M., Bargozini F. (2023). Vibration of a nanocomposite annular sandwich microplate based on HSDT using DQM. Multiscale Sci. Eng..

[192] Yuan Y., Zhao K., Han Y., Sahmani S., Safaei B. (2020). Nonlinear oscillations of composite conical microshells with in-plane heterogeneity based upon a couple stress-based shell model. Thin-Walled Struct..

[193] Yang Y., Sahmani S., Safaei B. (2021). Couple stress-based nonlinear primary resonant dynamics of FGM composite truncated conical microshells integrated with magnetostricitive layers. Appl. Math. Mech..

[194] Fan L., Sahmani S., Safaei B. (2021). Couple stress-based dynamic stability analysis of functionally graded composite truncated conical microshells with magnetostrictive facesheets embedded within nonlinear viscoelastic foundations. Eng. Comput..

[195] Suwankornkij P., Pulngern T., Tangbanjongkij C., Chucheepsakul S., Jiammeepreecha W. (2025). Static analysis of a hemispherical nanoshell under uniform pressure based on MCST: A comparison of FEM and GDQ solutions. Arch. Appl. Mech..

[196] Al-Furjan M.S.H., Habibi M., Ebrahimi F., Chen G., Safarpour M., Safarpour H. (2020). A coupled thermomechanical approach for frequency information of electrically composite microshell using heat-transfer continuum problem. Eur. Phys. J. Plus.

[197] Mohammadimehr M., Atifeh S.J., Rousta N.B. (2017). Stress and free vibration analysis of piezoelectric hollow circular FG-SWBNNTs reinforced nanocomposite plate based on modified couple stress theory subjected to thermo-mechanical loadings. J. Vibr. Control.

[198] Mao Y.H., Shang Y., Cen S., Li C.F. (2023). An efficient 3-node triangular plate elementfor static and dynamic analyses of microplates based on modified couple stress theory with micro-inertia. Eng. Comput..

[199] Wang S., Qian Z., Shang Y. (2024). Size-dependent finite element analysis of FGMs in thermal environment based on the modified couple stress theory. Eng. Comput..

[200] Dehrouyeh-Semnani A.M., Mostafaei H. (2021). Vibration analysis of scale-dependent thin shallow microshells with arbitrary planform and boundary conditions. Int. J. Eng. Sci..

[201] Soleimani I., Beni Y.T., Dehkordi M.B. (2019). Size-dependent two-node axisymmetric shell element for buckling analysis with couple stress theory. Proc. Instit. Mech. Eng. Part C J. Mech. Eng. Sci..

[202] Wang S.H., Shang Y., Qian Z.H. (2022). Size-dependent analysis of porous multi-directional FG shell structures based on the modified couple stress theory using the unsymmetric finite element method. Acta Mech..

[203] Nguyen T.C.N., Le M.H., Tran V.K., Nguyen T.D., Phung V.M. (2022). Static bending analysis of variable thickness microplates using the finite element method and modified couple stress theory. J. Sci. Technol..

[204] Korayem M.H., Hefzabad R.N. (2024). A quadrilateral non-classical microplate element considering the voltage effect. Proc. Instit. Mech. Eng. Part C J. Mech. Eng. Sci..

[205] Zhang B., He Y., Liu D., Gan Z., Shen L. (2013). A non-classical Mindlin plate finite element based on a modified couple stress theory. Eur. J. Mech. A/Solids.

[206] Taghizadeh M., Askari A.R., Farzinpoor H. (2024). Size-dependent finite element buckling analysis of porous cylindrical microshells reinforced by graphene platelets. Mech. Based Des. Struct. Mach..

[207] Genao F.Y., Kim J., Zur K.K. (2021). Nonlinear finite element analysis of temperature-dependent functionally graded porous microplates under thermal and mechanical loads. Compos. Struct..

[208] Karamanli A., Aydogdu M. (2020). Vibration of functionally graded shear and normal deformable porous microplates via finite element method. Compos. Struct..

[209] Wu H.P., Shang Y., Cen S., Li C.F. (2023). Penalty C^0^ 8-node quadrilateral and 20-node hexahedral elements for consistent couple stress elasticity based on the unsymmetric finite element method. Eng. Anal. Bound. Elem..

[210] Wang S., Qian Z., Shang Y. (2024). Size-dependent vibration analysis of porous 3D-FG microshells in complex thermal environments using a neural network enhanced finite element model. Case Stud. Therm. Eng..

[211] Thai T.Q., Zhuang X., Rabczuk T. (2021). A nonlinear geometric couple stress-based strain gradient Kirchhoff-Love shell formulation for microscale thin-wall structures. Int. J. Mech. Sci..

[212] Ansari R., Shojaei M.F., Mohammadi V., Bazdid-Vahdati M., Rouhi H. (2015). Triangular Mindlin microplate element. Comput. Methods Appl. Mech. Eng..

[213] Zuo D., Safaei B., Sahmani S., Ma G. (2022). Nonlinear free vibrations of porous composite microplates incorporating various microstructural-dependent strain gradient tensors. Appl. Math. Mech..

[214] Li L., Pan Y., Arabmarkadeh A. (2021). Nonlinear finite element study on forced vibration of cylindrical micro-panels based on modified strain gradient theory. Mech. Adv. Mater. Struct..

[215] Roque C.M.C., Ferreira A.J.M., Reddy J.N. (2013). Analysis of Mindlin micro plates with a modified couple stress theory and a meshless method. Appl. Math. Modell..

[216] Roque C.M.C., Zur K.K. (2022). On the static, vibration, and transient responses of micro-plates made of materials with different microstructures. Eng. Anal. Bound. Elem..

[217] Zhang Y., Sahmani S., Safaei B. (2022). Meshfree-based applied mathematical modeling for nonlinear stability analysis of couple stress-based lateral pressurized randomly reinforced microshells. Eng. Comput..

[218] Yang Z., Safaei B., Sahmani S., Zhang Y. (2022). A couple-stress-based moving Kriging meshfree shell model for axial postbuckling analysis of random checkerboard composite cylindrical microshells. Thin-Walled Struct..

[219] Liu H., Safaei B., Sahmani S. (2022). Combined axial and lateral stability behavior of random checkboard reinforced cylindrical microshells via a couple stress-based moving Kriging meshfree model. Arch. Civ. Mech. Eng..

[220] Liu S., Yu T., Bui T.Q., Xia S. (2017). Size-dependent analysis of homogeneous and functionally graded microplates using IGA and a non-classical Kirchhoff plate theory. Compos. Struct..

[221] Nguyen H.X., Atroshchenko E., Nguyen-Xuan H., Vo T.P. (2017). Geometrically nonlinear isogeometric analysis of functionally graded microplates with the modified couple stress theory. Comput. Struct..

[222] Nguyen H.X., Nguyen T.N., Abdel-Wahab M., Bordas S.P.A., Nguyen-Xuan H., Vo T.P. (2017). A refined quasi-3D isogeometric analysis for functionally graded microplates based on the modified couple stress theory. Comput. Methods Appl. Mech. Eng..

[223] Fan F., Xu Y., Sahmani S., Safaei B. (2020). Modified couple stress-based geometrically nonlinear oscillations of porous functionally graded microplates using NURBS-based isogeometric approach. Comput. Methods Appl. Mech. Eng..

[224] Thai C.H., Nguyen-Xuan H., Nguyen L.B., Phung-Van P. (2022). A modified strain gradient meshfree approach for functionally graded microplates. Eng, Comput..

[225] Hung P.T., Phung-Van P., Thai C.H. (2023). Small scale thermal analysis of piezoelectric-piezomagnetic FG microplates using modified strain gradient theory. Int. J. Mech. Mater. Des..

[226] Thai H.T., Vo T.P. (2013). A size-dependent functionally graded sinusoidal plate model based on a modified couple stress theory. Compos. Struct..

